# Neuroimmune communication: from CNS-resident cell networks to the glioblastoma microenvironment

**DOI:** 10.3389/fimmu.2026.2002570

**Published:** 2026-10-28

**Authors:** Yanfei Li, Qiuhong Zheng, Xianwu Wang, Huaian Fu, Jiancheng Chen, Lili Yao, Junyan Lin, Rui Wang

**Affiliations:** 1 Cell Therapy Research Center, Xiamen Humanity Hospital, Xiamen, China; 2 School of Medicine, Xiamen University, Xiamen, China; 3 Department of Radiology, The Second Affiliated Hospital of Xiamen Medical College, Xiamen, China

**Keywords:** nervous system, immune cells, glioma, glial cells, glioma-associated macrophages/microglia

## Abstract

Neurons, glial cells, and immune cells engage in extensive bidirectional communication within the central nervous system (CNS) through shared receptors, ligands, and secreted mediators. Glial cells — particularly astrocytes and microglia — serve not merely as structural support but as central immunomodulatory hubs that relay neural signals to immune effectors. In the context of CNS malignancies, glioblastoma (GBM) co-opts these neuroimmune networks to establish an immunosuppressive tumor microenvironment. This narrative review is organized around five interconnected axes. After summarizing the receptor and ligand repertoires of CNS-resident cell types, we examine (i) the neural inputs that reach glioblastoma and the reciprocal signals the tumor returns to the neural compartment; (ii) the remodeling of glial cells and glioma-associated macrophages/microglia; (iii) the innate and adaptive immune suppression that follows, including a “chemical–cellular” suppressive architecture; (iv) the blood–brain barrier and neurovascular interface that constrain immune access; and (v) the therapeutic opportunities arising from each of these layers.

## 1 Introduction

The human nervous and immune systems comprise a wide diversity of cell types, each exhibiting distinct functions under both health and disease conditions. The interactions between these two systems are exceptionally complex. Although numerous intriguing and significant discoveries have been made in the field of neuroimmunology in recent years, many unanswered questions remain, awaiting further investigation and exploration. This review focuses on the interactions among neurons, glial cells, immune cells, and tumor cells during the course of neurological disorders. We aim to elucidate and synthesize the underlying mechanisms, and to highlight the remaining challenges as well as potential therapeutic strategies that may be achievable in the future.

## 2 The CNS cellular substrate: neurons, glia and resident immune cells

Beyond its neuronal components, the central nervous system (CNS) comprises a functionally diverse population of glial cells that play essential roles in maintaining homeostasis, synaptic modulation, and circuit integrity. The physiological operation of the CNS relies not only on close neuron-glia interactions (1, 2) but is also dynamically regulated by resident and infiltrating immune cells, which collectively shape neural function in health and disease (3, 4).

### 2.1 Receptor repertoires of neurons

Neuronal cells express a diverse array of receptor types, which exhibit substantial heterogeneity in structure, function, and signaling mechanisms, thereby underpinning the complex information-processing capabilities of the nervous system. Based on current research, receptors predominantly expressed on the neuronal surface can be categorized into two major classes: ionotropic receptors and metabotropic receptors. These classes are further subdivided into multiple subfamilies, encompassing diverse ligand systems including neurotransmitters, neuropeptides, and growth factors.

Ionotropic receptors represent a class of protein complexes that form ligand-gated ion channels directly, whose activation rapidly alters the membrane potential and mediates fast synaptic transmission on a millisecond timescale. These receptors typically assemble as multimeric subunits arranged around a central pore. Upon binding of specific neurotransmitters, conformational changes induce channel opening, allowing selective transmembrane ion flux. Within the glutamatergic system, for example, α-amino-3-hydroxy-5-methyl-4-isoxazolepropionic acid receptors (AMPAR, GRIA family), N-methyl-D-aspartate receptors (NMDAR, GRIN family), and kainite receptors (GRIK family) all belong to the ionotropic receptor class. They are responsible for mediating rapid excitatory synaptic currents, voltage-dependent calcium influx, and transient depolarizing responses, respectively (5). Notably, NMDA receptors require both glutamate binding and membrane depolarization to relieve their Mg²^+^ block (6). In addition, γ-aminobutyric acid (GABA) mediates rapid inhibitory signaling by acting on GABA-A receptors (ligand-gated chloride channels composed of subunits such as GABRB and GABRA), thereby inducing postsynaptic hyperpolarization or shunting inhibition (5). Acetylcholine additionally activates nicotinic acetylcholine receptors (nAChRs), which form sodium- and potassium-permeable cation channels that contribute extensively to the regulation of attention, arousal, and reward circuitry (7).

Metabotropic receptors belong to the G protein–coupled receptor (GPCR) superfamily and act through heterotrimeric G proteins (Gs, Gi/o, Gq/11) to initiate second-messenger cascades, producing slower but sustained modulation. Prototypical examples include metabotropic glutamate receptors (mGluRs, such as GRM2/3), which regulate synaptic plasticity and network excitability through cAMP or IP3/DAG signaling pathways (5).Dopamine receptors (DRD1-5) are classified into D1-like (Gs-coupled) and D2-like (Gi/o-coupled) families based on their associated G proteins, which respectively stimulate or inhibit cAMP production and thereby profoundly influence motor control, motivated behavior, and mental states (5). The majority of serotonin receptor subtypes (e.g., 5-HT1A, 5-HT2A) also belong to this metabotropic class and are involved in regulating emotion, sleep, and cognitive functions (5). It is noteworthy that some receptors exhibit hybrid characteristics; for example, certain kainite receptors not only function as ligand-gated ion channels but can also activate G proteins through non-canonical pathways, illustrating the complexity of receptor signaling (8).

Beyond the classical neurotransmitter receptors, neurons express a diverse array of additional functional receptors. For instance, tropomyosin receptor kinase (Trk) family receptors (TrkA, TrkB, TrkC) and p75NTR specifically bind to nerve growth factor (NGF), brain-derived neurotrophic factor (BDNF)/neurotrophin-4(NT-4), and NT-3, respectively, regulating neuronal survival, axonal growth, and synaptic plasticity (9). In the purinergic receptor family, the P2X receptors (e.g., P2X4) function as ATP-gated cation channels and are implicated in processes such as pain perception and neuroinflammation (10). Notably, studies have detected NMDA receptor expression even in non-neuronal cell types such as lymphocytes, suggesting physiological roles beyond conventional synaptic transmission (11). Furthermore, neurons express numerous neuropeptide receptors, including opioid receptors, the substance P receptor (NK1R), and calcitonin gene-related peptide (CGRP) receptors, which participate in pain modulation, stress responses, and autonomic regulation (12). Single-cell transcriptomic analyses have resolved dozens of neuronal subtypes in the rodent cortex, each with a distinct receptor repertoire (6); this heterogeneity is relevant here because the receptor complement of the neurons adjacent to a tumor determines which neurotransmitter-driven mechanisms are available to it.

### 2.2 Glial cell populations

Recent advances in neuroimmunology have positioned the interactions between glial cells and immune cells in the brain as a major research cross field across both neuroscience and immunology (13, 14). Glial cells, which are numerically predominant and functionally diverse within neural tissue, constitute approximately 90% of all cells in the human CNS. The principal glial populations in the brain include astrocytes, microglia, and oligodendrocytes (15). Astrocytes are integral components of the neurovascular unit, a structural and functional module that also encompasses cerebral blood vessels, pericytes, and neurons (16). Astrocytes regulate vascular function and blood-brain barrier (BBB) integrity, control metabolic and redox homeostasis, and maintain electrolyte and water balance, thereby playing essential roles in preserving BBB integrity, modulating neurotransmitter equilibrium (17), and supporting neuronal metabolism (18). Under pathological conditions, astrocytes can propagate inflammatory responses that drive disease progression (19), restrict inflammatory cells infiltration into the CNS parenchyma (20), and provide trophic support and regulation for neuronal repair and regeneration (20–22).

Microglia possess a dual identity: they are a subtype of glial cells and simultaneously serve as the primary resident immune cells in the brain, functioning as myeloid tissue-resident macrophages derived from the embryonic yolk sac (23, 24). The processes of microglia remain in a state of continuous surveillance under physiological conditions. Upon brain injury, microglia rapidly become activated and respond (25, 26). They are equipped with the capacity to sense pathogen- and damage-associated signals, enabling them to monitor the local microenvironment and clear pathogens as well as cellular debris. Through mechanisms such as complement-mediated synapse elimination and synaptic pruning, microglia play crucial roles in neuronal circuit development. Genetic variants that lead to selective or elevated expression of multiple microglial genes are recognized as risk factors for neurodegenerative diseases (27, 28). Furthermore, microglia contribute to immune responses via cytokine secretion and phagocytic activity (29).

Oligodendrocytes are derived from the differentiation of oligodendrocyte progenitor cells (OPCs) (30) and play a central role in the formation and maintenance of myelin sheaths (31), thereby ensuring efficient propagation of nerve impulses (32). Emerging evidence suggests that oligodendrocytes may also participate in immune-related functions (33), potentially through direct interactions with neuronal soma (34) or via crosstalk with the cerebral vasculature (35).

Under physiological conditions, glial cells contribute to the maintenance of CNS homeostasis through dynamic interactions with neurons (36). In contrast, under pathological conditions, glial cells become activated and participate in inflammatory responses, playing critical roles in neuroinflammation and injury repair (37). Moreover, circulating immune cells that infiltrate the CNS can directly interact with both neurons and glial cells (38, 39).

### 2.3 Glial receptors and gliotransmitters

As previously noted, neurons represent the fundamental functional units of the nervous system, responsible for electrical signaling and neurotransmitter transmission. They form extensive synaptic connections, releasing neurotransmitters such as adrenaline, norepinephrine, dopamine, acetylcholine, and glutamate into the synaptic cleft, where they act on receptors of neighboring neurons. Additionally, the synaptic microenvironment contains various neurotrophic factors and chemokines-including brain‐derived neurotrophic factor (BDNF), hepatocyte growth factor (HGF), neurotrophin-3, C-X-C motif chemokine ligand 12 (CXCL12), and C-X3-C motif chemokine ligand 1 (CX3CL1) (40)—which exert their effects through direct or indirect interactions with surrounding cells. Notably, the expression of diverse receptors on glial cells provides a critical interface for these molecules to modulate neural function and signaling (**Table 1**).

**Table 1:** Summary of CNS cell types, key receptors, ligands, and primary functions.

Cell type	Receptor category	Key receptors	Major ligands	Primary functions
Neurons	Ionotropic	AMPAR, NMDAR, GRIK, GABAAR, nAChRs, P2X4	Glutamate, GABA, Acetylcholine, ATP	Fast synaptic transmission, inhibitory signaling (5–8, 10)
	Metabotropic	mGluRs, DRD1–5, 5-HT1A/2A, Opioid receptors, NK1R, CGRP-R	Glutamate, Dopamine, Serotonin, Neuropeptides	Synaptic plasticity, motor control, emotion, pain modulation (5, 12)
	Growth factor receptors	TrkA/B/C, p75NTR	NGF, BDNF, NT-3, NT-4	Neuronal survival, axonal growth, synaptic plasticity (9)
Astrocytes	Metabotropic (GPCRs)	Glutamate-R, GABAR, Adenosine-R, NE-R, AChR, Endocannabinoid-R	Glutamate, GABA, Adenosine, NE, ACh	Ca²^+^ mobilization, gliotransmitter release, BBB integrity, metabolic support (41–43)
	Secreted factors	—	ATP, D-serine, Thbs1/2, SPARC, Hevin, TNFα, CSPGs, ADNF, S100β	Synaptogenesis, AMPAR/NMDAR recruitment, neuroprotection (44–49, 66)
Microglia	Ionotropic/Metabotropic	iGluRs, mGluRs, GABAR, P2X/P2Y, Adenosine-R, AChR, NE-R, DA-R, Cannabinoid-R	Glutamate, GABA, ATP, Adenosine, ACh, NE, DA	Cytokine release, ROS production, phagocytosis, synaptic pruning, pain regulation (50–53)
Oligodendrocytes	Glutamate/Growth factor/Others	NMDAR, AMPAR, mGluR5, EPOR, NG2, DDR1, Integrins, Nogo-R, HCN2, LDL/VLDL-R	Glutamate, EPO, ECM components, Cholesterol	Myelination, myelin repair, neurite outgrowth regulation, cholesterol transport (54–65)

Astrocytes express a diverse repertoire of neurotransmitter receptors, which can be categorized by ligand specificity, including receptors for glutamate, GABA, adenosine, norepinephrine, acetylcholine, and endocannabinoids. Most of these receptors are G protein-coupled (41, 42) and capable of mobilizing intracellular calcium release. In addition to receptor-mediated signaling, astrocytes actively release a variety of neuromodulators—such as ATP, adenosine, glutamate, and D-serine (43)—which, together with classical neurotransmitters, are collectively referred to as gliotransmitters. Beyond these, astrocytes secrete a spectrum of other signaling molecules that fulfill distinct regulatory roles. For instance, thrombospondins (Thbs1/2) and SPARC (secreted protein acidic and rich in cysteine) modulate processes including cell adhesion, migration, angiogenesis, and immune function (44, 45). Secreted protein hevin and cholesterol support the induction of structural synapse formation and maturation (46, 47). Molecules like glypican-4/6, tumor necrosis factor-α (TNFα), and chondroitin sulfate proteoglycans (CSPGs) contribute to the recruitment of AMPA receptors to postsynaptic membranes, thereby facilitating fast excitatory synaptic transmission essential for learning and memory (48, 49). Moreover, activity-dependent neuroprotective factor (ADNF) promotes the clustering of NMDA receptors at synaptic sites, further shaping synaptic function and plasticity (45).

Similar to neurons, microglial neurotransmitter receptors can be broadly categorized into ionotropic and metabotropic types (50). Ionotropic glutamate receptors (iGluRs), for example, can interact with TNF-α, while metabotropic glutamate receptors (mGluRs) play significant roles in both neurotoxicity and neuroprotection (51). Classified by transmitter, microglia also express GABA receptors, whose activation can enhance interleukin (IL) release (e.g., IL-8), as well as purinergic receptors that, upon ligand engagement, regulate cell migration and cytokine production (52, 53). In addition, microglia possess receptors for adenosine, acetylcholine, norepinephrine, dopamine, neuropeptides, opioids, and cannabinoids (50, 51). Ligand binding to these diverse receptors enables microglia to modulate key functions such as cytokine and free radical release, potassium and calcium flux, as well as their own activation, motility, and involvement in pain regulation (50) (52, 53).

Oligodendrocytes express several key receptors, including NMDA receptors (54), AMPA receptors (55), and mGluR5 receptors (56), which belong to the glutamate receptor family and are critically involved in excitatory neurotransmission, excitotoxicity, and myelin growth. They also express erythropoietin receptors (EPOR) (57) and the nerve/glia antigen 2 (NG2) proteoglycan receptor (58), both of which function as neurotrophic/cytokine receptors that directly or indirectly regulate oligodendrocyte proliferation and differentiation upon ligand binding. Additionally, oligodendrocytes possess a range of other receptors such as adhesion molecules (integrins) (59), the extracellular matrix receptor discoidin domain receptor 1 (DDR1) (60, 61), the tight-junction protein claudin-11, Nogo receptors (62), hyperpolarization-activated cyclic nucleotide–gated 2 (HCN2) channels (63), LDL/VLDL receptors (64), and calcium-signaling related receptors (65). These molecules play important roles in myelin formation and repair, modulation of autoimmune responses, regulation of neurite outgrowth, cholesterol uptake and transport, and other key physiological processes.

Neuroglial cells communicate with neurons through the release of gliotransmitters, thereby modulating neuronal information processing. These gliotransmitters bind to receptors on the postsynaptic neuronal membrane, leading to alterations in neuronal membrane potential. Dendrites, the primary structures for signal reception in neurons, express voltage-gated channels such as hyperpolarization-activated cyclic nucleotide–gated (HCN) channels and A-type potassium channels, which fine-tune the dendritic response to gliotransmitter-mediated signaling (66). Additionally, glial cells express connexins, which form channels permitting the passage of bioactive molecules smaller than 1 kDa and are implicated in various neurological disorders (67). Both activated glia and neuronal dendrites harbor numerous voltage-gated ion channels (VGICs) and exhibit calcium-induced calcium release (CICR) mechanisms. These plasma membrane channels engage in intricate interactions with calcium released from the endoplasmic reticulum, shaping intracellular calcium dynamics. S100β, a protein secreted predominantly by glial cells, binds extracellular free calcium, thereby reducing its concentration and consequently alleviating the inhibitory effect of extracellular calcium on persistent sodium channels in the neuronal membrane (66).

### 2.4 Reactive glial programmes co-opted by tumors

Following nerve injury, astrocytes in the peri lesional area typically secrete bioactive substances that facilitate tissue repair at the injury site (22) (**Figure 1**). However, upon microglial activation, released factors such as TNF, IL-1α, and C1q can drive astrocytes toward a dysfunctional state, which impedes neuronal recovery (68). Studies have shown that microglia promote the shift of astrocytes toward a neuroprotective phenotype and stimulate scar formation by downregulating the P2Y1 purinergic receptor. When this activity is inhibited, astrocytes exhibit reduced scar‐forming capacity, which is detrimental to the healing process after traumatic brain injury (TBI) (69). In investigations of ionizing radiation effects on glial cells, irradiated microglia become reactive and release IL-1β and TNFα, thereby promoting irradiation‐induced astrogliosis (70, 71). In the experimental autoimmune encephalomyelitis (EAE) model, microglia‐derived TGFα signals to astrocytes and attenuates disease severity, whereas microglia-derived vascular endothelial growth factor β (VEGFβ) exacerbates the pathology (72); tryptophan metabolites modulate this process via the aryl hydrocarbon receptor (AHR) (72). A recent study revealed that reactive microglia predominantly cluster around vasopressin-secreting neurons, where they phagocytose astrocytic elements and prune astrocytic processes. This leads to increased glutamate spillover, elevated neuronal activity, and enhanced vasopressin release, ultimately contributing to the development of hypertension (73). These reactive programmes are introduced here only insofar as they constitute the baseline glial repertoire that glioblastoma subsequently co-opts; the general neuroinflammatory literature is not reviewed exhaustively.

**Figure 1:**
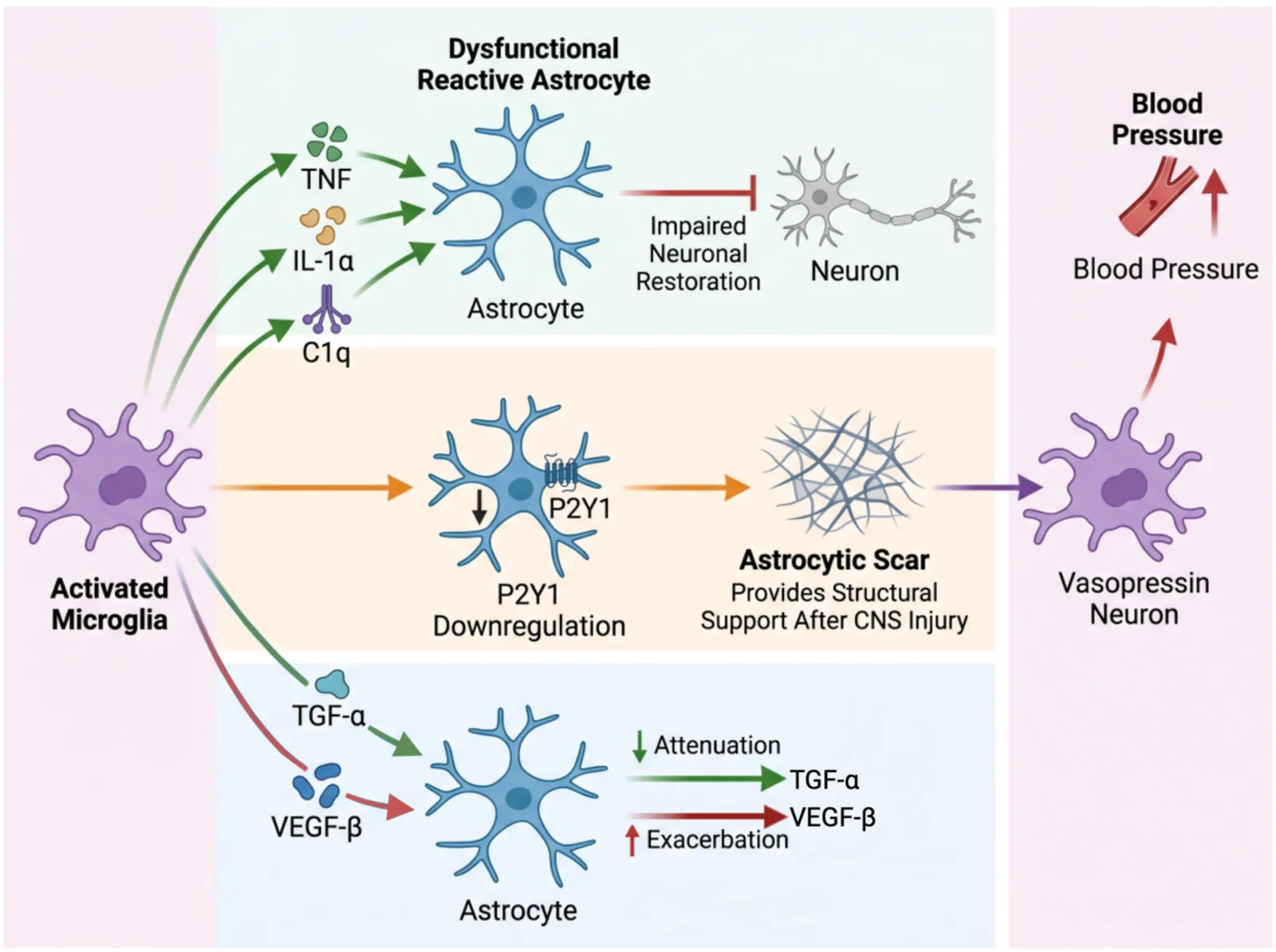
The interplay involved in astrocytes. (C1q, complement 1q; IL-1α, interleukin-1α; TGF, tumor growth factor; TNF, tumor necrosis factor; VEGF, vascular endothelial growth factor β).

Oligodendrocytes, the principal components of neuronal myelin sheaths, receive signals from microglia that critically regulate their differentiation and survival (74, 75) (**Figure 2**). Specifically, the modulation by CD11c-expressing microglia plays a pivotal role in myelination and neural repair (76). Both *in vivo* and *in vitro* studies indicate that under physiological conditions, microglia help maintain a stable pool of oligodendrocyte precursor cells (77). Upon microglial activation, such as during inflammation, the release of TNF, nitric oxide, and complement components promotes oligodendrocyte death (78, 79), followed by their subsequent phagocytosis by microglia. In contrast, both IL-13 and IL-10 can drive microglial toward an anti-inflammatory, repair-supportive activation state, which supports neural repair and regeneration (80, 81). Moreover, IL-10 cooperatively interacts with neurotrophin-3 (NT-3) to enhance myelination and improve functional recovery (82). Together, these findings indicate that both cytokines promote oligodendrocyte survival and differentiation by promoting this reparative microglial state (83). Conversely, the absence of CD206 impairs the function of reparative macrophages/microglia, thereby hindering oligodendrocyte differentiation (83).

**Figure 2:**
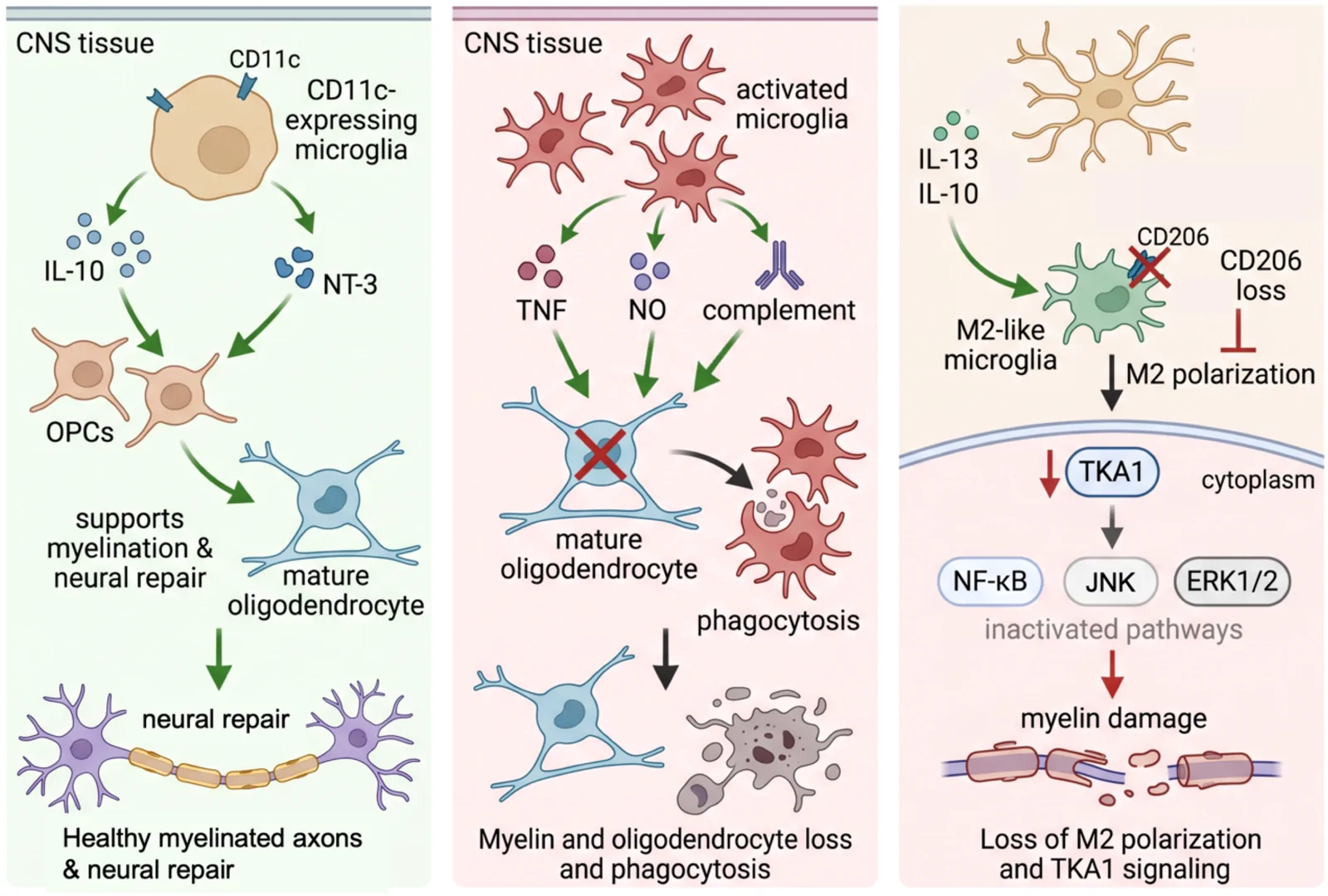
The interplay involved in oligodendrocytes. (ERK, extracellular signal-regulated kinase, iNOS, inducible nitric oxide synthase, JNK, Jun N-terminal kinase, NF-κB, nuclear factor kappa-B, NT-3, neurotrophin-3, NO, nitric oxide, TAK1, TGFβ-activated kinase 1).

Additionally, extracellular matrix (ECM) influences oligodendrocyte differentiation and survival (84), with the adhesion G protein‐coupled receptor (ADGRG1) serving as a key regulator of oligodendrocyte development (85), and transglutaminase-2 acting as its important ligand (85). In studies of experimental autoimmune encephalomyelitis (EAE), loss of TGFβ-activated kinase 1 (TAK1) in microglia suppresses inflammation through the nuclear factor kappa-B (NF-κB), Jun N-terminal kinase (JNK), and extracellular signal-regulated kinase 1 (ERK1)/ERK2 pathways (86), thereby reducing myelin damage. Furthermore, research has shown that human microglia *in vitro* can directly kill neural progenitor cells, leading to a decline in oligodendrocyte numbers (87).

## 3 Neural inputs to glioblastoma

A terminological distinction is applied throughout this section: “neural signals” denotes neurotransmitters, neuropeptides and neurotrophins released by neurons or by nerve-associated glia, whereas mediators of the same chemical identity synthesized by tumor cells or stromal cells are designated as tumor- or stroma-derived and act through autocrine or paracrine loops. The two sources are not interchangeable—they differ in spatial distribution, in whether release is activity-dependent, and consequently in which therapeutic interventions are applicable. The neural signals that promote tumor growth can be broadly categorized into direct and indirect mechanisms (**Table 2**).

**Table 2:** Neural signals promoting tumor growth: direct and indirect mechanisms.

Mode	Signal/source	Tumor receptor/target	Downstream pathway	Tumor effect	References
Direct					
	Glutamate	NMDAR	GKAP → FMRP/HSF1; Ca²^+^ → CaMKII/MAPK → CREB; CREB → METTL3 → HK2	Tumor invasion; glycolysis ↑; PNI	(88–90)
		AMPAR	MAPK/K-Ras	Tumor invasion and migration	(91)
	Acetylcholine	—	ACh → PD-L1 ↑ on tumor cells	Immune evasion from CD8^+^ T cells	(92)
		CHRM1	EGFR/MAPK/PI3K/AKT ↓	Tumor suppression	(94)
		CHRM3	Autocrine/paracrine loop	Tumor growth and proliferation ↑	(94)
		CHRM4	NGF → CHRM4 → AKT	Migration, invasion, dissemination	(95)
		CHRNA5	Ca²^+^ → CaMKII/GSK-3β/β-catenin	Metastasis and drug resistance ↑	(96)
	GABA	GABABR	GSK-3β/β-catenin ↓	Tumor immune escape	(93)
	Catecholamines	ADRB2	ETV1/c-KIT → MAPK-ERK → AKT; Beclin1/VPS34 → HIF-1α	GIST progression; p53 degradation; autophagy impairment	(97–102)
		ADRB3	Epac/JNK → BDNF; mTOR → G0/G1 transition	BDNF release ↑; cell cycle promotion	(103)
	Neurotrophins	p75NTR, TrkB, TrkC	ERK/AKT	Tumor invasiveness ↑	(104, 105)
Indirect					
	NE (neurons)	ADRB2 on vascular endothelial cells	Tumor neovascularization; ADRB2/PAK/Src/HIF-1α/c-Myc → hTERT ↑	Angiogenesis ↑; EMT promotion	(40, 106, 107)
	MDK (neurons)	CD8^+^ T cells → CCL4; Microglia → CCL5	CCL4 ↑ in CD8^+^ T cells → insufficient anti-tumor response; CCL5 → AKT/GSK3β/CREB	Anti-tumor T cell response ↓; tumor growth and survival ↑	(108, 109)
	Schwann cells	Tumor cells (TASTs formation)	c-Jun → reprogramming → TASTs; CCL2 → monocyte recruitment → cathepsin B^+^ macrophages	Tumor cell dissemination; PNI enhancement	(40, 110)
		Pancreatic cancer cells	IL-6 → STAT3	Metastasis and invasion ↑	(111)
		CXCR2 on lung cancer cells	CXCL5 → CXCR2 → PI3K/AKT/GSK-3β → Snail/Twist	EMT ↑; metastatic potential ↑	(112)
	Tumor microtubes	Glioma cells	GAP43 and Ttyh1-dependent microtube formation	Proliferation ↑; invasiveness ↑; radioresistance ↑	(113)

### 3.1 Direct neurotransmitter–receptor signaling to tumor cells

Tumor cells express NMDARs and AMPARs, which can mediate downstream signaling upon binding to glutamate released from presynaptic membranes (**Figure 3**). Following glutamate binding, NMDAR activation promotes the Fragile X Messenger Ribonucleoprotein (FMRP)/heat shock factor 1 (HSF1) signaling pathway via guanylate kinase-associated protein (GKAP), thereby driving tumor invasion. Additionally, NMDAR-mediated calcium influx activates the Ca²^+^/calmodulin-dependent protein kinase II (CaMKII)/mitogen-activated protein kinase (MAPK) pathway, leading to phosphorylation of cAMP response element-binding protein (CREB) and enhanced transcriptional activity (88). CREB activation induces expression of methyltransferase-like 3 (METTL3), which upregulates hexokinase 2 (HK2) expression, enhances glycolysis, and promotes perineural invasion (PNI) (89). NMDAR signaling also activates downstream pathways involving CaMK and the mitogen-activated extracellular signal-regulated kinase (MEK) –MAPK cascade, ultimately resulting in CREB activation that facilitates tumor growth and invasion (90). Similarly, AMPAR engagement by glutamate activates the MAPK/K- rat sarcoma virus (Ras) pathway, further supporting tumor progression (91).

**Figure 3:**
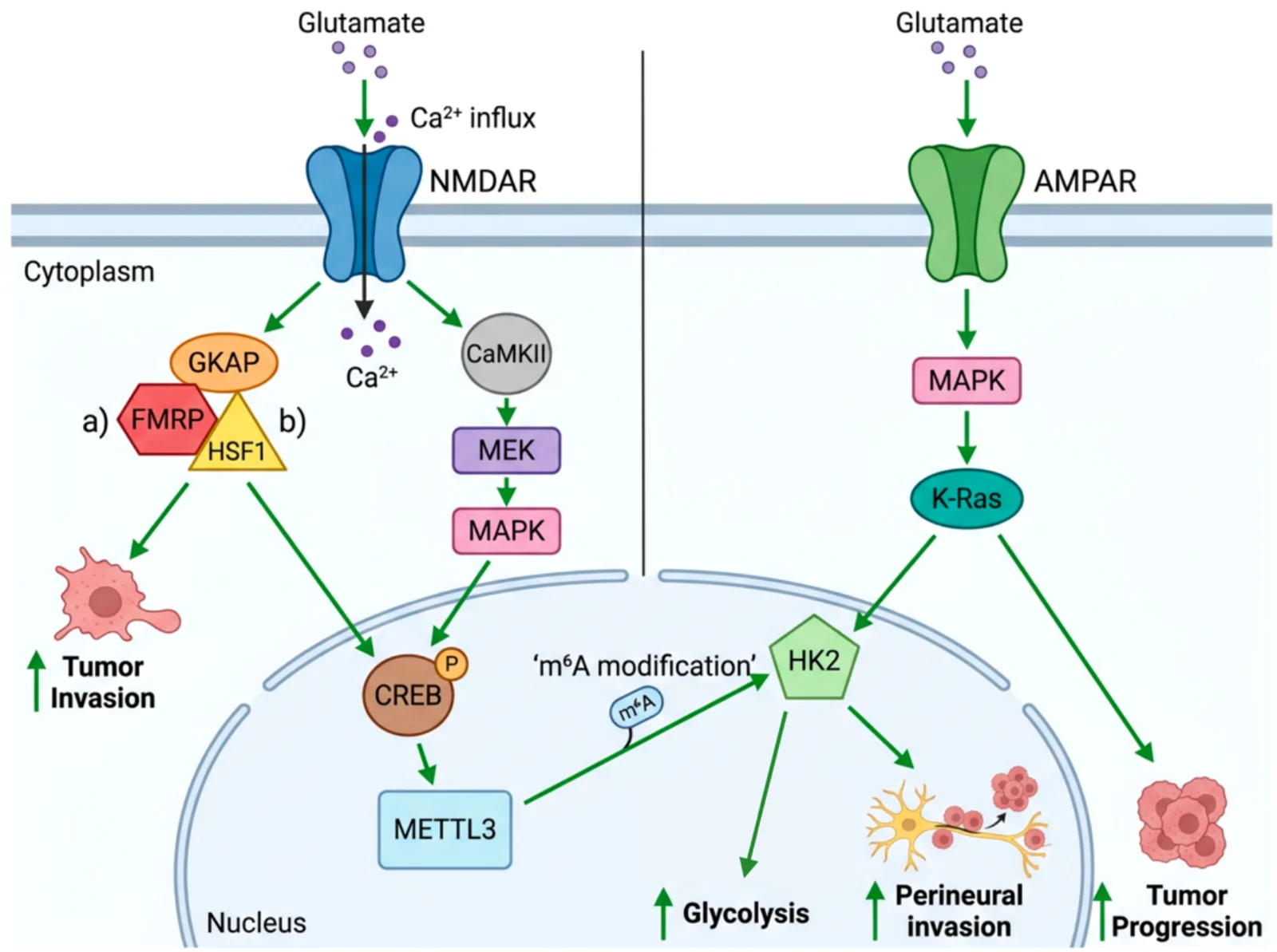
The signal transduction of glutamate on tumor. (CREB, cAMP response element-binding protein; FMRP, Fragile X Messenger Ribonucleoprotein; GKAP, guanylate kinase-associated protein; HK2, hexokinase 2; HSF1, heat shock factor 1; MAPK, mitogen-activated protein kinase; MEK, mitogen-activated extracellular signal-regulated kinase; METTL3, methyltransferase-like 3).

Acetylcholine upregulates programmed cell death 1 ligand 1 (PD-L1) expression in tumor cells, thereby enabling immune evasion from CD8+T cell-mediated cytotoxicity (92). γ-aminobutyric acid (GABA) activates the GABA-B receptor, leading to inhibition of glycogen synthase kinase 3β (GSK-3β) and β-catenin activity, which promotes tumor immune escape (93). Upon binding of acetylcholine released from presynaptic terminals to cholinergic receptors on tumor cells, a cascade of downstream responses is initiated. Activation of cholinergic receptor muscarinic 1 (CHRM1) suppresses the epidermal growth factor receptor (EGFR)/MAPK/phosphatidylinositol 3-kinase (PI3K)/protein kinase B (AKT) pathway, thereby inhibiting tumor progression. Acetylcholine synthesized by the tumor cells themselves—a tumor-derived rather than neuron-derived source—can stimulate their own growth and proliferation through an autocrine or paracrine loop via muscarinic acetylcholine receptor 3 (CHRM3) (94). NGF induces CHRM4 expression and activates the AKT signaling pathway, facilitating tumor cell migration, invasion, and dissemination (95). Furthermore, acetylcholine binding to the α5 nicotinic acetylcholine receptor subunit (CHRNA5) activates the Ca^2+^-dependent CaMKII/GSK-3β/β-catenin signaling axis, and the subsequent upregulation of β-catenin enhances tumor metastasis and drug resistance (96).

Presynaptic release of catecholamines activates beta-2 adrenergic receptors (ADRB2), thereby promoting the ETS translocation variant 1 (ETV1)/c-KIT signaling axis, which drives progression in gastrointestinal stromal tumors (GIST) (97–99). In GIST, ETV1 enhances tumor growth via MAPK-ERK (extracellular signal-regulated kinase) signaling and also activates AKT, leading to ubiquitination-mediated degradation of p53 and promoting tumor cell survival (100). Furthermore, ADRB2-mediated upregulation of AKT disrupts the Beclin1/vacuolar protein sorting 34 (VPS34)/Atg14 complex, impairing autophagy and stabilizing hypoxia-inducible factor 1α (HIF-1α); these changes collectively enhance tumor cell adaptation to hypoxia and stress (101, 102). The activation of beta-3 adrenergic receptors (ADRB3) promotes tumor progression through two distinct mechanisms: first, via the exchange protein activated by cAMP (Epac)/c-Jun N-terminal kinase (JNK)-dependent pathway, increasing brain-derived neurotrophic factor (BDNF) release from tumor cells; second, by stimulating the transition from G0 to G1 phase through the mammalian target of rapamycin (mTOR) pathway (103). These adrenergic mechanisms have been characterized largely in peripheral tumors such as GIST, and their transferability to GBM is partial: ADRB2 is expressed by glioma cells and by GAMs, and the sympathetic–ADRB2–cAMP/PKA axis is operative in the GBM microenvironment, whereas the ETV1/c-KIT module is lineage-restricted to GIST and has no established counterpart in glioma. We therefore cite these studies as mechanistic precedents for adrenergic control of tumor behavior, not as evidence obtained in GBM.

Additionally, neurotrophins including BDNF, NGF, and NT-3 can activate tumor cell receptors p75 neurotrophin receptor (p75NTR), tropomyosin receptor kinase B (TrkB), and tropomyosin receptor kinase C (TrkC), respectively, enhancing tumor cell invasiveness via the ERK/AKT signaling pathway (104, 105).

### 3.2 Indirect neural inputs: vascular, stromal and nerve-associated routes

Neurons can release norepinephrine, which activates ADRB2 on vascular endothelial cells to promote tumor neovascularization (106, 107). Furthermore, norepinephrine upregulates human telomerase reverse transcriptase (hTERT) expression via the ADRB2/p21-activated kinase (PAK)/Src proto-oncogene (Src)/HIF-1α and c-Myc signaling axis, thereby facilitating epithelial-mesenchymal transition (EMT) (40).

Neurons can also produce the neurotrophic factor Midkine (MDK), which induces the expression of the pro inflammatory chemokine C-C motif ligand 4 (CCL4) in CD8+ T cells. This process contributes to an insufficient anti-tumor T-cell response within the tumor microenvironment. Additionally, CCL4 promotes the release of CCL5 from microglia, thereby activating the AKT/GSK-3β/CREB signaling pathway and further supporting tumor growth and survival (108, 109).

Schwann cells undergo reprogramming via c-Jun-dependent pathways, subsequently enveloping tumor cells to form tumor-activated Schwann cell trajectories (TASTs), which facilitate tumor cell dissemination (110) (**Figure 4**). Activated Schwann cells secrete C-C motif chemokine ligand 2 (CCL2), recruiting monocytes that differentiate into cathepsin B-expressing macrophages and thereby enhancing tumor perineural invasion (PNI) (40). *In vitro* evidence further demonstrates that Schwann cell-derived interleukin-6 (IL-6) promotes pancreatic cancer cell metastasis and invasion through the signal transducer and activator of transcription 3 (STAT3) signaling pathway (111). Additionally, Schwann cell-secreted C-X-C motif chemokine ligand 5 (CXCL5) interacts with C-X-C chemokine receptor type 2 (CXCR2) on lung cancer cells, activating the PI3K/AKT/GSK-3β axis and EMT markers such as Snail and Twist, ultimately increasing cancer cell metastatic potential (112). Perineural invasion and Schwann cell reprogramming are properties of tumors growing along peripheral nerves and have been demonstrated in pancreatic, lung and head-and-neck cancer; the CNS parenchyma contains no Schwann cells, so these specific mechanisms do not operate in GBM. They are included here because the underlying principle—that tumors co-opt nerve-associated glia to build invasive conduits—has a direct CNS analogue in the astrocyte- and microglia-supported perivascular and white-matter invasion of glioma, and because the chemokine axes involved (CCL2, CXCL5/CXCR2, IL-6/STAT3) are also active in the GBM microenvironment.

**Figure 4:**
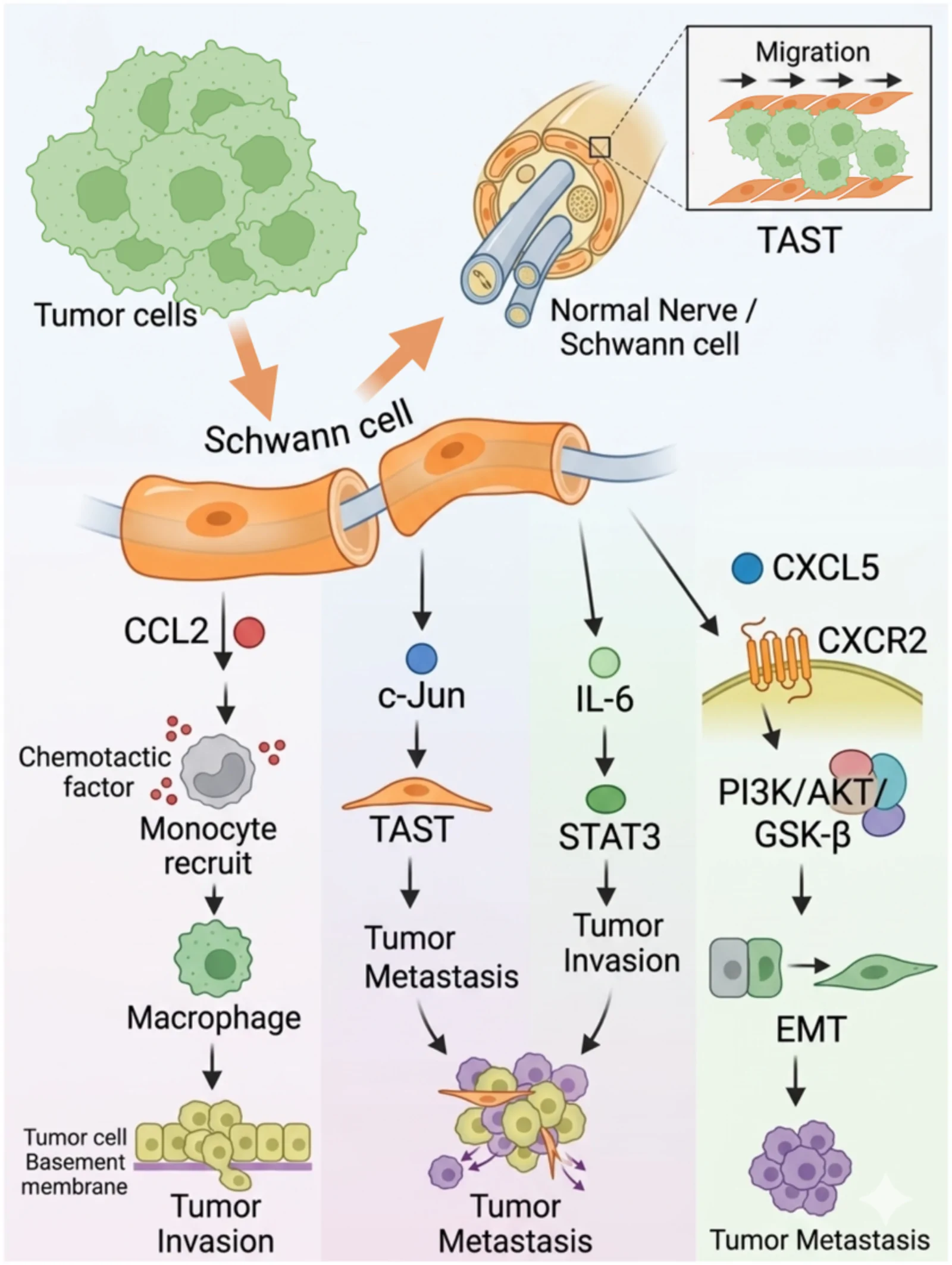
Schwann cells and tumor promotion. (AKT, protein kinase B; EMT, epithelial-mesenchymal transition; GSK-3β, glycogen synthase kinase 3β; PI3K, phosphatidylinositol 3-kinase; STAT3, signal transducer and activator of transcription 3; TASTs, tumor-activated Schwann cell trajectories).

The formation of certain tumor microtubes depends on growth‐associated protein 43 (GAP-43) and tweety-homolog 1 (Ttyh1). These microtubes enhance microtubule‐dependent proliferation, invasiveness, and radio resistance in glioma cells (113). The preceding subsections considered signals travelling from the nervous system to the tumor; the communication is, however, bidirectional, and the reciprocal arm is considered next.

### 3.3 Reciprocal tumor-to-neuron signaling and network integration

First, glioma cells act on glial cells in a paracrine manner to modulate their secretory profiles. In turn, these glia-derived factors feed back onto glioma cells, altering their phenotype. Within this process, glial cells may upregulate factors such as insulin-like growth factor-binding protein 2 (IBP-2), tissue inhibitor of metalloproteinase 2 (TIMP-2), fibronectin (FN), myeloid-derived growth factor (MYDGF), and SPARC-like protein 1 (SPARCL1), thereby enhancing their own activity and activating ERK, AKT, and JNK signaling pathways, which collectively reduce cell death and suppress glioma cell migration (114) (**Figure 5**). Second, tumor cells interact with astrocytes via molecules associated with tumor microtubes (TMs), including gap junction proteins such as Connexin 43, as well as Thrombospondin-1 and ROCK, thereby compromising therapeutic efficacy. Through gap junctions, glioma cells deliver cyclic GMP-AMP (cGAMP) to astrocytes, establishing a pro-inflammatory milieu characterized by the robust release of cytokines, particularly interferon-α (IFN-α) and TNF. These cytokines subsequently activate STAT1 and NF-κB pathways in tumor cells (115), promoting metastatic behavior. Gap junction-mediated exchange of small molecules such as Ca2+, ATP, and metabolic toxins also contributes to cellular homeostasis, further supporting tumor growth and invasion (116) (117) (**Figure 5**). Furthermore, during tumor progression, varying degrees of axonal damage occur. Astrocytes and microglia jointly facilitate axonal regeneration, a process termed reactive gliosis, wherein reactive astrocytes release substantial quantities of cytokines that may inadvertently promote brain tumor growth. Cancer metastasis leads to the release of Serpins, which counteract plasmin produced by the reactive stroma. Consequently, FasL signaling on astrocytes is disrupted, potentially facilitating further cancer dissemination (118). Finally, the interplay between glioma and neuronal cells is mediated by synaptic and paracrine pathways. Synaptic signaling operates primarily through AMPA receptors, while paracrine signaling is facilitated by post-synaptic proteins such as neuroligin-3 (NLGN-3), BDNF, and IGF-1. These signals induce activation of the PI3K pathway and upregulation of FOS expression in glioma cells, which in turn feedback to enhance NLGN-3 expression, thereby promoting cellular proliferation and the formation of new synapses (116) (119) (**Figure 5**).

**Figure 5:**
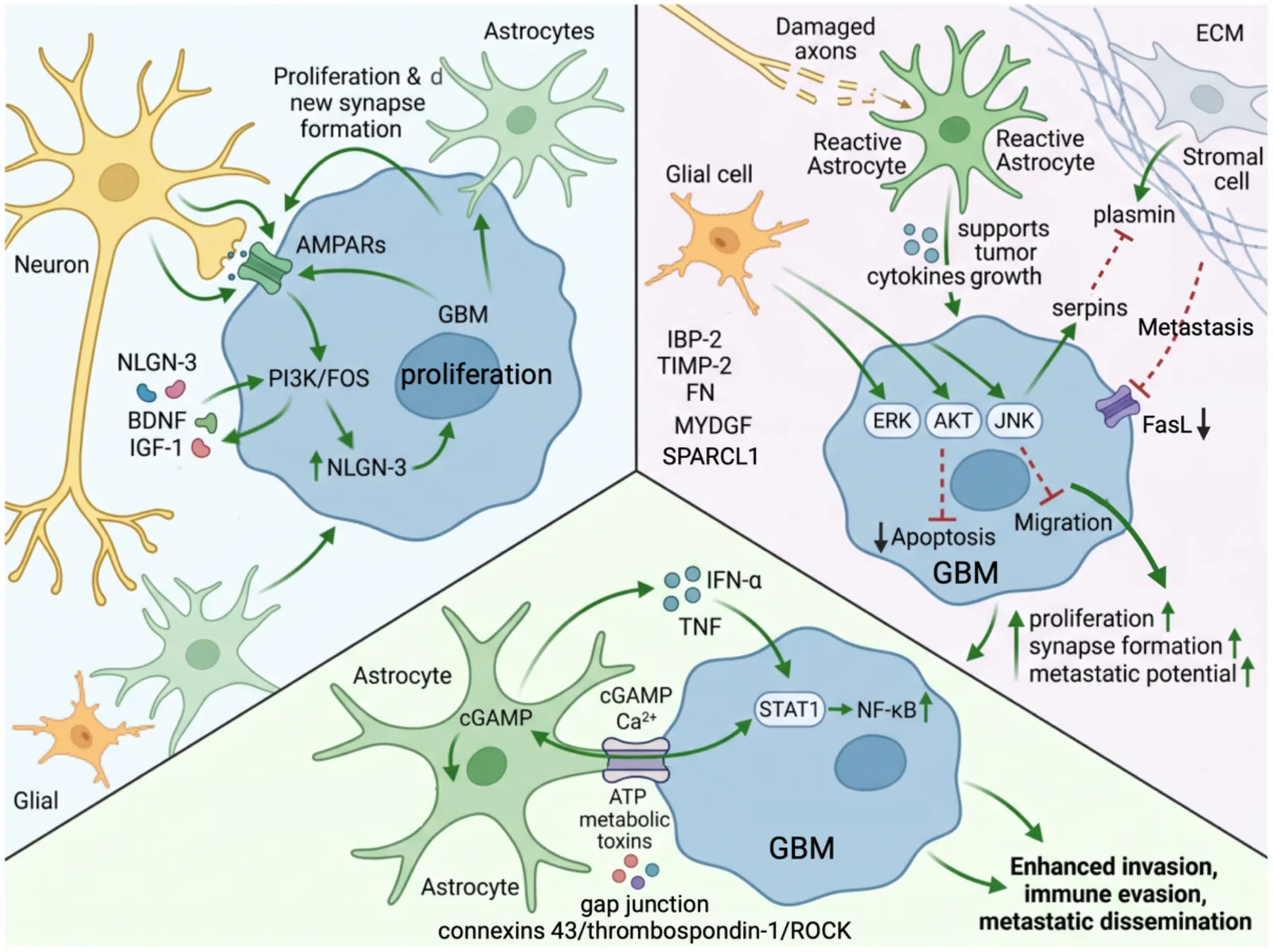
Tumor cells disturb glial and neurons. (BDNF, brain-derived neurotrophic factor; ECM, extracellular matrix; FN, fibronectin; IBP-2, insulin-like growth factor-binding protein 2; IGF-1, insulin-like growth factor; MYDGF, myeloid-derived growth factor; NLGN-3, neuroligin-3; ROCK, Rho-associated coiled-coil containing protein kinase; SPARCL1, SPARC-like protein 1; TIMP-2, tissue inhibitor of metalloproteinase 2).

## 4 Glial and GAM remodeling of the glioblastoma microenvironment

Glioma, the most common primary malignant tumor of the CNS, is characterized by a highly complex tumor microenvironment that plays a pivotal role in tumor initiation, progression, and immune evasion (120, 121). This microenvironment encompasses not only neoplastic cells but also a diverse array of non-neoplastic cellular components, including endothelial cells (122), pericytes (123), microglia/macrophages (124, 125), and astrocytes (126). These stromal elements engage in dynamic crosstalk with glioma cells, collectively shaping the malignant phenotype and driving tumor aggressiveness. In this network, interactions between glial cells and immune cells are particularly critical, involving multiple immunosuppressive and immune‐escape mechanisms that sustain tumor growth and therapeutic resistance.

### 4.1 Recruitment and ontogeny of glioma-associated macrophages/GAM

Tumor-associated macrophages (TAMs) refer to all macrophages infiltrating the tumor microenvironment, regardless of their origin from circulating monocytes or tissue-resident populations. They represent the most abundant immune cell population within the tumor stroma. In glioblastoma multiforme (GBM), microglia, as tissue-resident macrophages, outnumber those derived from peripheral blood monocytes and are specifically termed glioma-associated macrophages/microglia (GAMs). GAMs can constitute more than 50% of the total tumor mass in GBM (127), and their extent of infiltration correlates significantly with tumor malignancy (128). In the central nervous system (CNS), normal glial cells, macrophages, and glioma cells interact via inflammatory mediators and their receptors (**Table 3**). Throughout this review we therefore reserve “GAM” for the glioma-resident myeloid compartment as a whole and use “TAM” only when referring to tumor-associated macrophages in non-CNS tumors or to the general concept; where the distinction is mechanistically relevant, microglia-derived and monocyte-derived GAMs are named explicitly, since the two populations differ in ontogeny, spatial distribution, transcriptional state and T cell–suppressive capacity (129–131).

**Table 3:** Bidirectional interactions between GAMs/TAMs and GBM.

Direction	Key mediators	Signaling pathway	Functional outcome	References
GBM → GAM recruitment	LOX, WISP1, CCL5/CXCL1, CCL2/TNFα, CXCL12, MCP-1/3, GDNF	PI3K/AKT, SRC, YAP1, WNT/β-catenin, NF-κB; PI3K–YAP1–LOX–PYK2	GAM infiltration into GBM microenvironment	(127, 132–135)
GAM → GBM promotion	Pleiotrophin, Osteopontin, VEGF, Oncostatin M, EGF, TGF-β, MMP-2, IL-6, IL-1β, CCL8	AKT; STAT3 → EMT; STAT3 → MIC-1/IL-10/IL-4/TGF-β	Tumor growth, angiogenesis, invasion, stem cell renewal	(136–144)
GBM → GAM immunosuppressive polarization	CSF-1, CXCL16, IL-4/IL-4R → IGF-1	CSF-1R; CXCR6; PI3K	Immunosuppressive phenotype shift; phagocytic activity ↓; tumor proliferation ↑	(140, 157, 165, 166)
GBM → Immunosuppression	TGF-β, IL-10, CD47–SIRPα, PD-L1	T cell apoptosis; CD8^+^ T cell inhibition; DC dysfunction; “don’t eat me” signal	Anti-tumor immune response ↓; immune evasion	(150–155)

Initially, the inactivation of tumor suppressors (e.g., phosphatase and tensin homolog (PTEN), NF1, TP53) in GBM cells leads to the dysregulation of multiple signaling pathways (including PI3K/AKT, SRC, YAP1, WNT/β-catenin, and NF-κB) (**Figure 6**). These pathways drive the secretion of various soluble factors (such as lysyl oxidase (LOX), WISP1, CCL5/CXCL1, and CCL2/TNFα), which recruit GAMs into the GBM microenvironment (127) and modulate anti-tumor responses. Additionally, other mediators, including CXCL12 acting through the SDF-1/CXCL12 axis (132), further recruit GAMs to hypoxic and invasive tumor regions. Molecules such as monocyte chemoattractant protein 1 (MCP-1), MCP-3 (133), glial cell-derived neurotrophic factor (134), and lysyl oxidase (LOX) (135) also contribute to GAM recruitment. Notably, the upregulation of LOX is mediated via the PI3K–YAP1 (Yes-associated protein 1)–LOX–PYK2 (proline-rich tyrosine kinase 2) signaling axis in GBM cells, a process that promotes macrophage infiltration and tumor progression (135).

**Figure 6:**
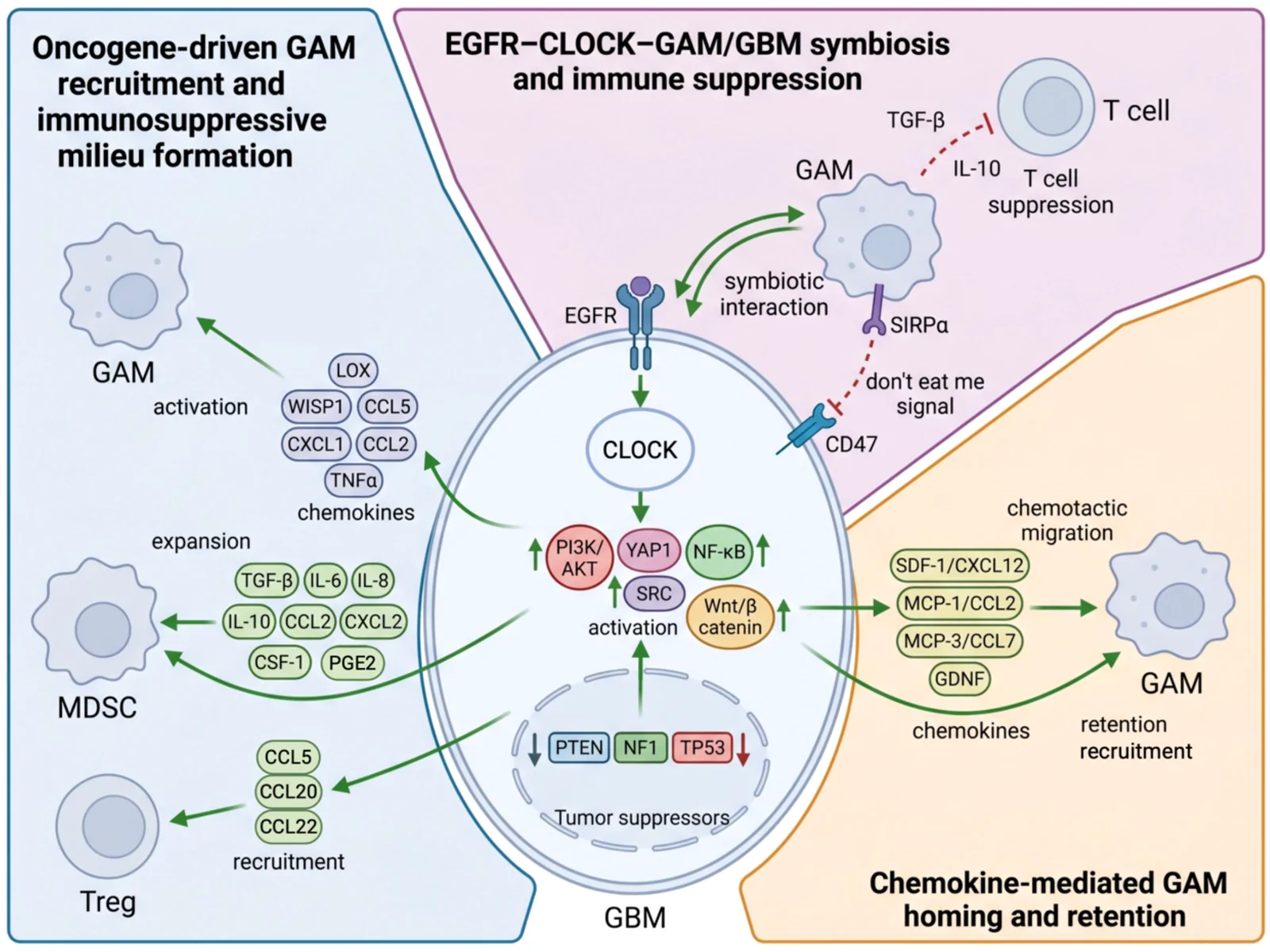
Overall GAM/TAM-GBM interaction. (AKT, protein kinase B; CCL, C-C motif chemokine ligand; CD47, cluster of differentiation 47; CLOCK, circadian locomotor output cycles kaput; CSF-1, colony-stimulating factor 1; CXCL, C-X-C motif chemokine ligand; EGFR, epidermal growth factor receptor; GAM, glioma-associated macrophages/microglia; GBM, glioblastoma; GDNF, glial cell-derived neurotrophic factor; IL, interleukin; LOX, lysyl oxidase; MCP, monocyte chemoattractant protein; MDSC, myeloid-derived suppressor cells; NF-κB, nuclear factor-kappa B; NF1, neurofibromin 1; PGE2, prostaglandin E2; PI3K, phosphatidylinositol 3-kinase; PTEN, phosphatase and tensin homolog; SDF-1, stromal cell-derived factor 1; SIRPα, signal regulatory protein α; SRC, Src proto-oncogene; STAT, signal transducer and activator of transcription; TGF-β, transforming growth factor beta; TNFα, tumor necrosis factor alpha; TP53, tumor protein p53; Treg, regulatory T cell; VEGF, vascular endothelial growth factor; WISP1, WNT1-inducible signaling pathway protein 1; Wnt, Wingless/Integrated; YAP1, Yes-associated protein 1).

GAMs subsequently secrete a variety of factors that promote glioma progression (**Figure 7**). For instance, pleiotrophin activates the AKT signaling pathway in glioma cells, thereby enhancing tumor growth and stem cell renewal (136). Osteopontin and vascular endothelial growth factor (VEGF) contribute to glioma angiogenesis (137). Macrophage-derived oncostatin M can induce a mesenchymal-like transcriptional program in glioblastoma via the STAT3 pathway (138), promoting epithelial–mesenchymal transition (EMT). Additional factors, including stress-inducible protein 1 (139), epidermal growth factor (EGF) (140), transforming growth factor-β (TGF-β), matrix metallopeptidase-2 (MMP-2) (141, 142), IL-12, IL-1β, IL-6 (127), and CCL8 (143), further drive GBM cell proliferation and invasion. Notably, IL-6 and IL-1β activate the STAT3 signaling pathway in GBM cells, leading to the release of macrophage inhibitory cytokine-1 (MIC-1), IL-10, IL-4, and TGF-β (144–149).

**Figure 7:**
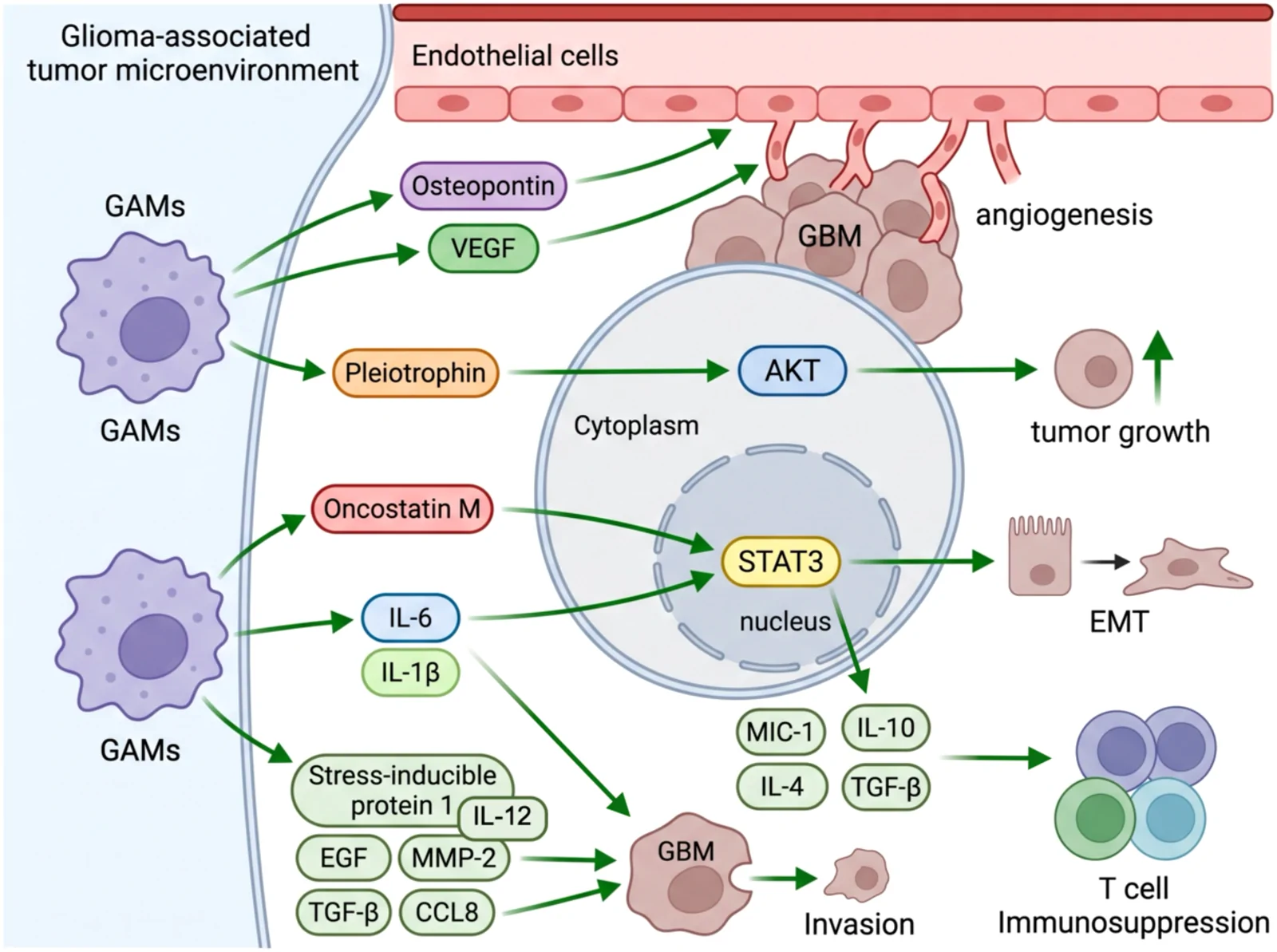
GAM promote glioma. (AKT, protein kinase B; CSF1:colony-stimulating factor 1; EGF, epidermal growth factor; GAM, glioma-associated macrophages/microglia; EMT, epithelial-mesenchymal transition; GBM, glioblastoma; MCP, monocyte chemoattractant protein; MDSC, myeloid-derived suppressor cells; MIC, macrophage inhibitory cytokine; MMP, matrix metallopeptidase; PGE2, prostaglandin E2; SDF1, stromal cell-derived factor 1; SIRPα, signal regulatory protein α; PTEN, phosphatase and tensin homolog; SRC, Src proto-oncogene; STAT, signal transducer and activator of transcription; YAP1, Yes-associated protein 1; VEGF, vascular endothelial growth factor).

Concurrently, activation of oncogenes such as EGFR and CLOCK in GBM enhances the adhesion, migration, and selective polarization of GAMs (**Figure 6**). These interactions establish a symbiotic relationship between GAMs and GBM cells, which continuously fuels tumor progression (127). Over time, as the tumor evolves, the tumor microenvironment reshapes both glial cells and GAMs, gradually impairing their anti-tumor functions (116). Transcriptional reprogramming of glial cells and macrophages leads to altered morphology and metabolism, driving the microenvironment toward an anti-inflammatory state. These cells increasingly adopt an immunosuppressive phenotype, secreting factors such as TGF-β and IL-10 (150), which promote T cell apoptosis, inhibit the activation and cytotoxicity of CD8+ T cells (151), and impair the antigen-presenting capacity of dendritic cells (152). Furthermore, studies indicate that CD47 expressed on glioma cells engages SIRPα on macrophages, delivering a “don’t eat me” signal that suppresses macrophage phagocytic function (153–155).

GBM also induces the recruitment and activation of immunosuppressive cells, such as myeloid-derived suppressor cells (MDSCs) and regulatory T cells (Tregs), through the secretion of various cytokines and chemokines (156, 157) (**Figure 6**). Evidence indicates that the peripheral blood of glioma patients contains higher levels of MDSCs compared to healthy individuals (158). These MDSCs comprise heterogeneous populations, including immature macrophages with immunosuppressive transcriptional profiles, granulocytes, and dendritic cells. Factors secreted by GBM—such as IL-6, IL-8 (159), IL-10, colony-stimulating factor 1 (CSF-1), CCL2 (160), CXCL2 (161), prostaglandin E2 (PGE-2), and TGF-β—collectively promote the migration of MDSCs into the glioma tumor microenvironment (162). In contrast, Treg cells (detailed later) are primarily recruited by chemokines including CCL5, CCL20, and CCL22 (160) (163, 164).

### 4.2 Immunosuppressive polarization and neural modulation of GAMs

GBM promotes the polarization of GAMs toward immunosuppressive activation states. The differentiation and survival of these immunosuppressive macrophages depend on colony-stimulating factor 1 (CSF-1) secreted by GBM, which also directly enhances tumor invasion (140). Pharmacological inhibition of CSF-1 signaling reduces the expression of immunosuppressive macrophage markers (e.g., CSF-1R), suppresses pro-tumor functions, and attenuates glioma growth and invasiveness. Under these conditions, elevated levels of granulocyte-macrophage colony-stimulating factor (GM-CSF) and IFN-γ drive the repolarization of GAMs toward a pro-inflammatory, tumoricidal state, enhancing their phagocytic activity against glioma cells (157). Furthermore, when tumor microenvironmental cues upregulate the pro-inflammatory IL-4/IL-4R axis in GAMs, increased secretion of insulin-like growth factor 1 (IGF-1) activates the PI3K pathway in tumor cells, thereby promoting proliferation (165). Tumor-derived CXCL16 also contributes to the pro-tumor phenotypic shift of GAMs by engaging its receptor CXCR6 on these cells (166). It should be emphasized that the M1/M2 nomenclature used here is a historical simplification. Single-cell RNA sequencing and CITE-seq of human and murine glioma have shown that GAMs do not segregate into two discrete states but instead occupy a continuum of transcriptional programmes, with subsets defined by lipid handling, hypoxia, interferon response and antigen presentation that are not captured by M1/M2 markers (129, 130). These states are further structured by ontogeny and anatomical location: microglia-derived GAMs predominate in newly diagnosed tumors and localize preferentially to the peritumoral brain, whereas monocyte-derived GAMs are enriched in the hypoxic, perinecrotic core, expand at recurrence, and exert stronger T cell–suppressive activity (129–131). Comparative profiling of gliomas and brain metastases indicates that these programmes are additionally shaped by tumor origin and IDH mutational status (167). In the discussion that follows, “M2-like” should therefore be read as shorthand for an immunosuppressive transcriptional programme rather than as a discrete cell identity (**Figure 8**).

**Figure 8:**
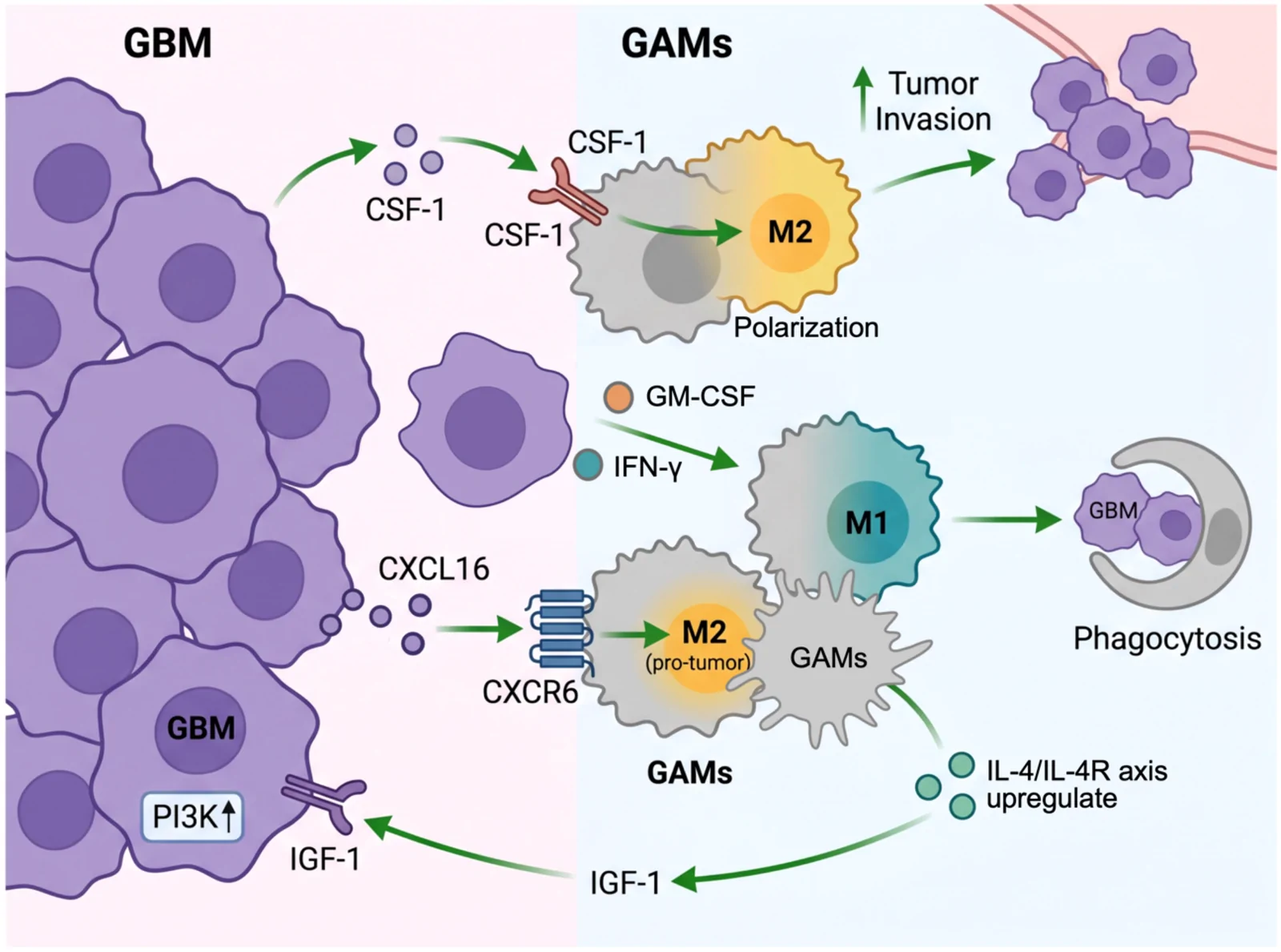
GAM transition. (CSF, colony-stimulating factor; EGF, epidermal growth factor; EMT, epithelial‐mesenchymal transition; GM-CSF, granulocyte-macrophage colony-stimulating factor; IGF, insulin-like growth factor; MIC-1, macrophage inhibitory cytokine-1; MMP-2, matrix metallopeptidase-2).

## 5 Innate and adaptive immune suppression in glioblastoma

### 5.1 Lymphocyte dysfunction: T cell exhaustion, Tregs and NK cells

T lymphocytes, including CD4+ helper T (Th) cells and CD8+ cytotoxic T lymphocytes (CTLs), constitute approximately 1–5% of total cells within the glioma microenvironment (168, 169). In non-tumor-invaded brain regions, TLR2-driven innate immune responses in normal glial cells can enhance the activation of CD8+T cells (170), thereby promoting anti-tumor cytotoxicity primarily through the release of perforin- and granzyme-containing granules. Th1 cells contribute indirectly to tumor control via secretion of cytokines such as IL-2, TNF-α, and IFN-γ (171, 172). To evade T cell–mediated immunity, glioma cells upregulate programmed cell death ligand 1 (PD-L1), which engages programmed cell death protein 1 (PD-1) on T cells and inhibits their differentiation and activation. The glioma tumor microenvironment also recruits Tregs through mediators including CCL2 (160) (173) and indoleamine 2,3-dioxygenase 1 (IDO1) (174). Recruited Tregs suppress immune responses by secreting inhibitory cytokines (e.g., IL-10, IL-35, TGF-β) (160) (175, 176), consuming IL-2 (177), and facilitating the conversion of ATP to adenosine, thereby impairing T cell activation (178). Consequently, T cells exhibit increased expression of PD-1 and cytotoxic T-lymphocyte–associated protein 4 (CTLA-4). Engagement of these receptors by PD-L1 on tumor cells drives T cell exhaustion (179–181). Furthermore, sympathetic nerve–derived norepinephrine acts on ADRB2 receptors expressed by Tregs, activating the cAMP/protein kinase A (PKA) signaling pathway and promoting Treg migration into the tumor microenvironment, where they exert immunosuppressive functions. Studies indicate that TGF-β secreted by Tregs inhibits immune responses via the Smad2/3 pathway, downregulating cell cycle proteins (e.g., cyclin D/E) while upregulating inhibitors such as p21 and p27, thereby arresting T cell proliferation (182). TGF-β also stabilizes Foxp3 expression, driving the differentiation of naïve CD4^+^ T cells into Tregs and inducing further secretion of IL-10, IL-35, and TGF-β, establishing a positive feedback loop. Additionally, TGF-β suppresses natural killer (NK) cell activity by downregulating NKG2D expression (151). Treg-expressed cytotoxic T-lymphocyte–associated protein 4 (CTLA-4) binds CD80/CD86 on antigen-presenting cells, leading to recruitment of SHP2 phosphatase and subsequent inhibition of T cell receptor (TCR) signaling, further dampening T cell activation (183). Collectively, these mechanisms attenuate anti-tumor immunity and facilitate glioma immune escape.

Beyond the PD-1/PD-L1 and CTLA-4 axes discussed above, several additional surface molecules restrain anti-tumor immunity in GBM and are relevant to the ATP-to-adenosine conversion described earlier. B7-H3 (CD276) is expressed in roughly three-quarters of GBM specimens and in patient-derived neurospheres but is largely absent from normal brain, and its expression correlates with malignancy grade and shorter survival; the tumor-restricted 4IgB7-H3 isoform is currently being pursued as a CAR-T target (184–186). T cell immunoglobulin and mucin domain-3 (TIM-3) and lymphocyte activation gene-3 (LAG-3) mark tumor-infiltrating T cells at successive stages of exhaustion, are detectable in the majority of human GBM samples, and their blockade improves survival in murine glioma when combined with anti-PD-1 or radiotherapy (187, 188). Finally, the ectonucleotidases CD39 (ENTPD1) and CD73 (NT5E) catalyze the sequential hydrolysis of extracellular ATP to AMP and then to adenosine, completing the pathway by which Tregs dampen T cell activation. CD39 is induced on GAMs through the aryl hydrocarbon receptor, while mass cytometry and single-cell RNA sequencing identified a CD73-high macrophage population that is specific to GBM among five tumor types and persists after anti-PD-1 treatment; CD73 deletion prolongs survival in mice receiving combined anti-PD-1 and anti-CTLA-4, nominating CD39/CD73 blockade as a rational addition to checkpoint therapy in GBM (189, 190).

Studies have demonstrated that glioma cells evade immune surveillance by downregulating MHC class I (MHC-I) expression (191, 192). There exists a close link between MHC-I expression and type I IFN signaling in glioma. Inhibition of protein phosphatase 2A (PP2A) in glioblastoma leads to the accumulation of cytosolic dsDNA, which activates the cGAS–STING pathway. This in turn enhances phosphorylation of interferon regulatory factor 3 (IRF3), promotes type I IFN production, activates the JAK/STAT1 signaling axis, and ultimately upregulates MHC-I expression (193) (**Figure 9**).

**Figure 9:**
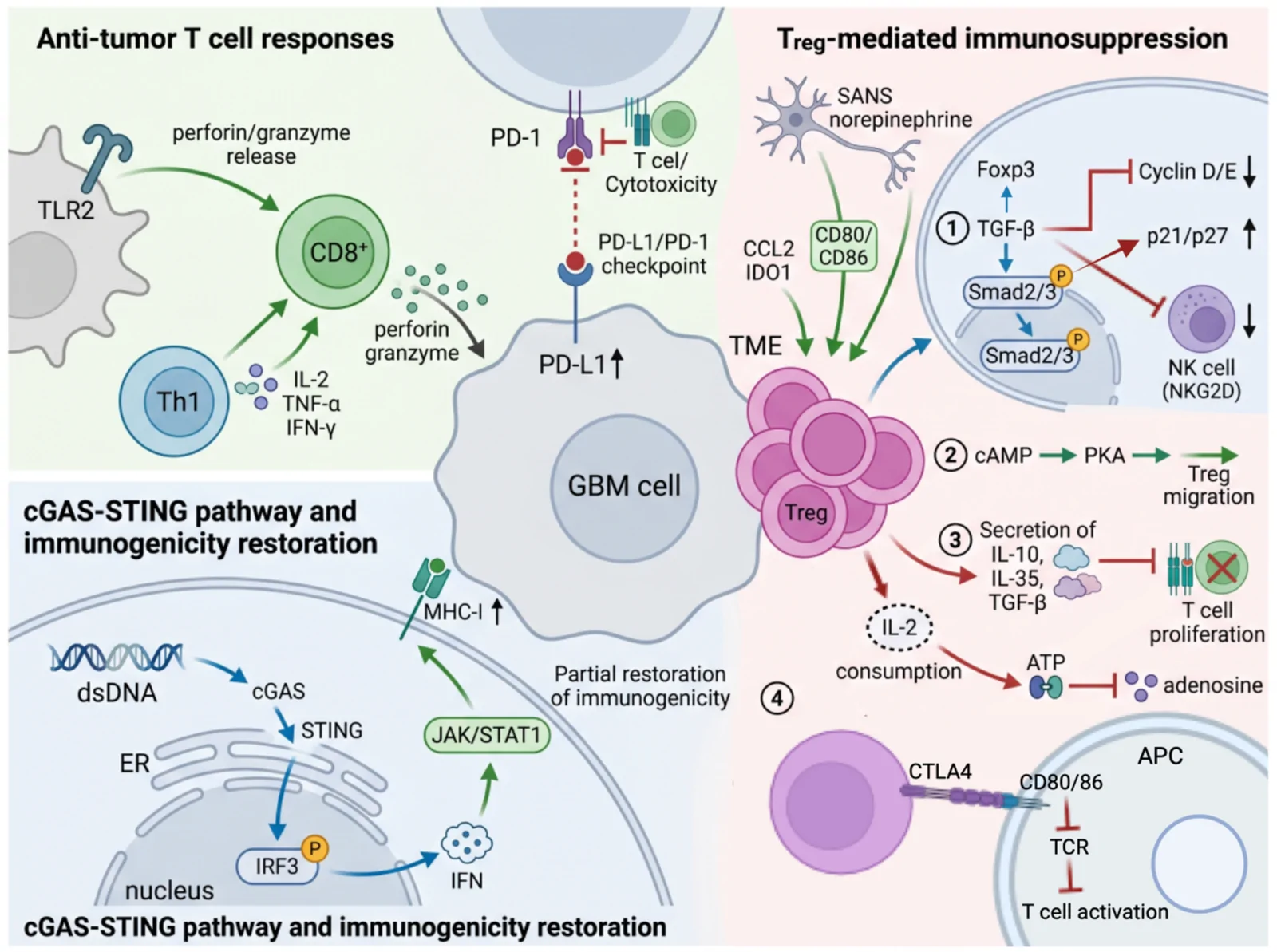
Lymphocytes work on CNS tumor. (APC, antigen presenting cell, cGAS-STING, cyclic GMP-AMP synthase-stimulator of interferon genes, CTLA4, cytotoxic T-lymphocyte-associated antigen-4, ER, endoplasmic reticulum, Foxp3, Forkhead box protein 3, IDO1, indoleamine 2,3-dioxygenase 1, IRF, interferon regulatory factor, JAK/STAT1, Janus kinase/signal transducer and activator of transcription 1, PKA, protein kinase A, SANS, sympathetic autonomic nervous system).

### 5.2 Neutrophils and dendritic cells

During the progression and metastasis of GBM, neutrophils exert their functions dependent on S100 family proteins (S100A4, S100A8, S100A9), which can induce mesenchymal transformation (194) of glioma cells. Glioma cells secrete granulocyte-colony stimulating factor (G-CSF) and granulocyte-macrophage colony-stimulating factor (GM-CSF) to promote neutrophil proliferation (195). Furthermore, under stimulation by neutrophil extracellular trap (NET)-derived IL-1, TNF-α, and high-mobility group box 1 (HMGB1) (196), glioma cells express IL-8 (197–199), which is responsible for neutrophil recruitment. Infiltration of intertumoral neutrophils holds prognostic significance (200), as increased neutrophils—both systemically and within the tumor microenvironment—predicts poorer clinical outcomes. Under chronic stress conditions, sustained sympathetic activation engages β-adrenergic receptors on tumor cells, enhancing the synthesis of tumor-derived exosomes and specificity protein 1(SP1). These subsequently activate neutrophils via TLR4, triggering the TLR4/NF-κB pathway and inducing IL-1β expression in neutrophils. Subsequently, IL-1β promotes tumor cell metastasis through two distinct pathways: an EGFR-dependent PI3K/AKT axis and an IL-1R-dependent MEK/ERK axis (201, 202). The former, via EGFR-dependent PI3K/AKT signaling, ultimately downregulates E-cadherin expression, promotes cytoskeletal reorganization, and upregulates vimentin expression, collectively increasing tumor cell motility and metastatic propensity. The latter, through the MEK/ERK pathway, ultimately drives epithelial–mesenchymal transition (EMT), thereby enhancing the migration and invasion (203) (**Figure 10**).

**Figure 10:**
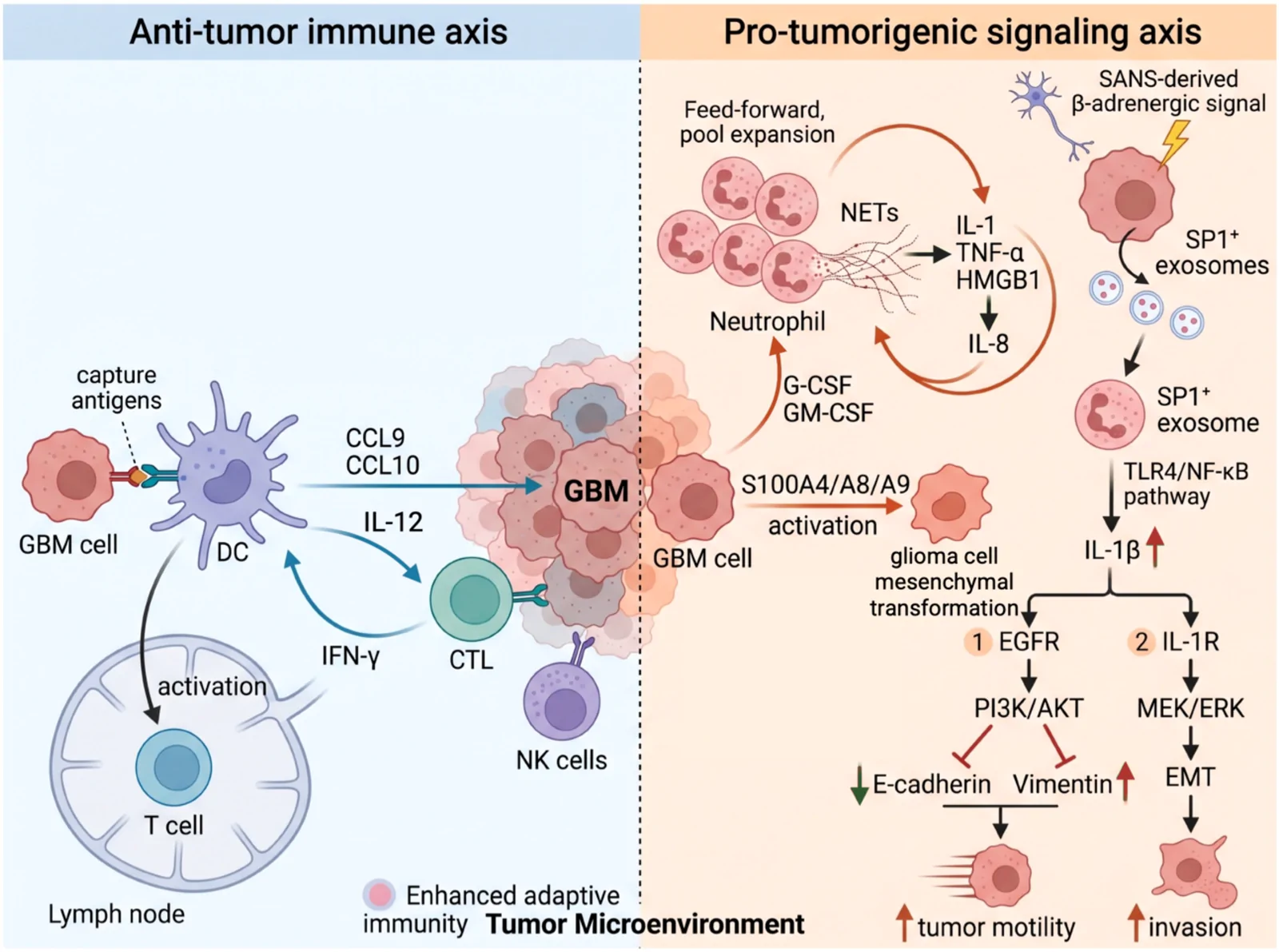
Effect of neutrophil and DC on CNS tumor. (EMT, epithelial-mesenchymal transition; ERK, extracellular signal-regulated kinase; HMGB, High mobility group box; MEK, mitogen-activated protein kinase kinase; NET, neutrophil extracellular trap; SP1, specificity protein 1).

Dendritic cells (DCs) are infrequently observed within the normal brain parenchyma but are relatively enriched at specific anatomical sites such as the choroid plexus and meninges (204, 205). When tumor cells colonize these locations, tumor-associated antigens can be captured by local DCs and subsequently transported via the intracranial lymphatic drainage system to deep cervical lymph nodes, where they are presented to T cells (206–208). Furthermore, DCs secrete chemokines including CCL9 and CCL10, which recruit cytotoxic T lymphocytes (CTLs) into the tumor microenvironment (208, 209). These infiltrating CTLs release IFN-γ, which in turn stimulates DCs to produce IL-12, thereby amplifying the anti-tumor responses mediated by T cells and natural killer (NK) cells (**Figure 10**).

### 5.3 Metabolic, exosomal and matrix-mediated immunosuppression

Glioblastoma cells further reshape the tumor microenvironment through metabolic reprogramming, generating metabolites that functionally impair immune cells (210). First, both glioma cells and astrocytes avidly uptake glucose and glutamine, leading to lactate accumulation and subsequent acidosis in the microenvironment (211). This acidic milieu suppresses T cell metabolic activity and inhibits IFN-γ secretion (212, 213). Second, glial cells highly express indoleamine 2,3-dioxygenase 1 (IDO1), which catabolizes tryptophan to generate L-kynurenine (Kyn). Tryptophan depletion by IDO1 recruits and activates Tregs while inducing apoptosis in effector T cells (189, 214–216). Concurrently, Kyn downregulates the expression of activating receptors NKG2D and NKp46 on natural killer (NK) cells (189, 214) (217), thereby suppressing their cytotoxic function. Third, glioma cells release cholesterol oxidation products that promote the polarization of GAMs toward an immunosuppressive state (218, 219). Moreover, hypoxia resulting from rapid glioma proliferation alters the metabolism of myeloid-derived suppressor cells (MDSCs), driving a reliance on fatty acid oxidation. This metabolic shift facilitates the upregulation of arginase-1 (Arg1) and enhances nitric oxide (NO) production, collectively exacerbating immunosuppression within the tumor microenvironment (220) (**Figure 11**).

**Figure 11:**
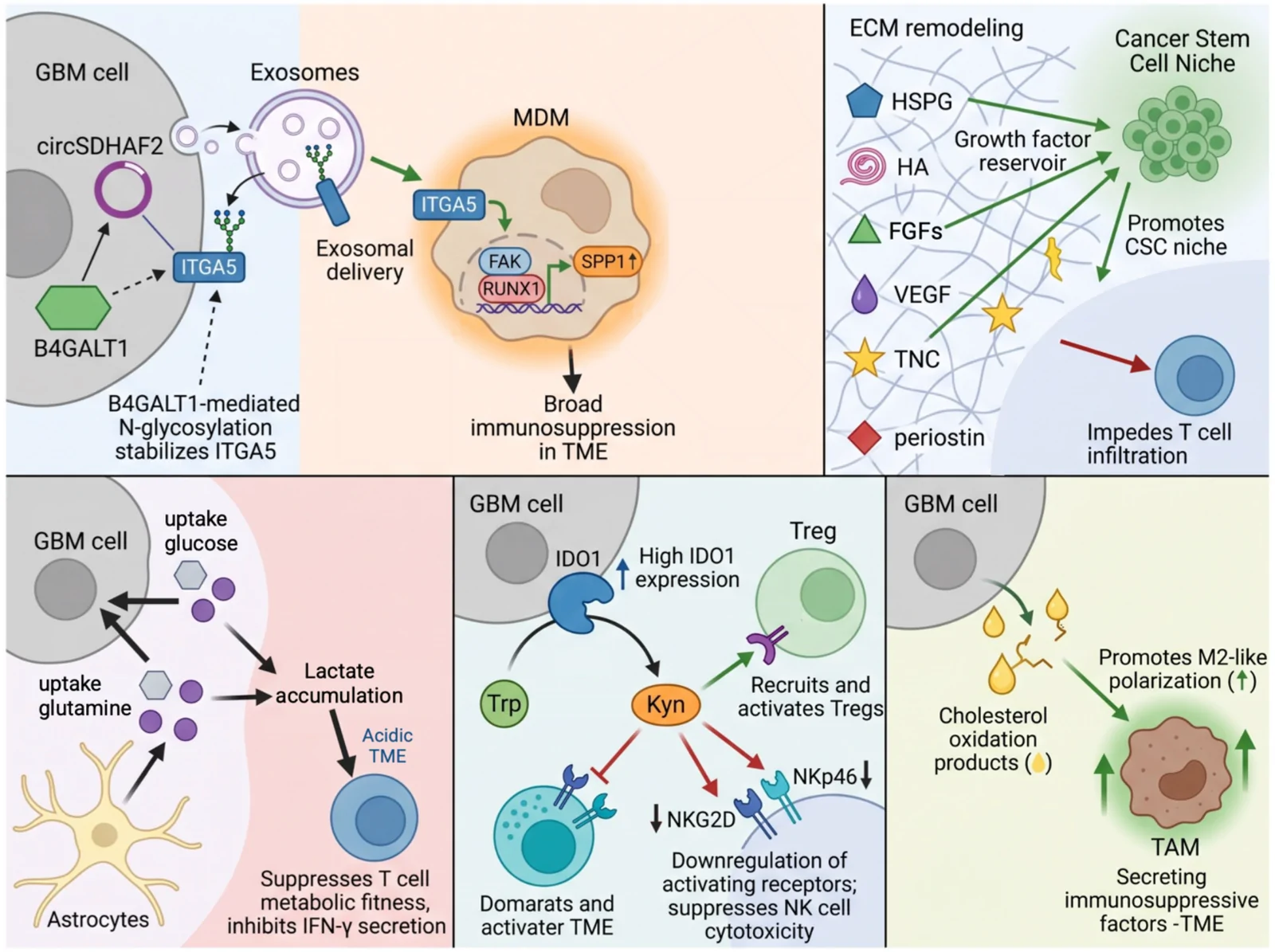
Metabolite, exosomes and ECM in CNS TME. (B4GALT1, β4-galactosyltransferase-1; circSDHAF2, circular RNA succinate dehydrogenase complex assembly factor 2; FAK, focal adhesion kinase; FGFs, fibroblast growth factor; HA, hyaluronic acid; HSPG, heparan sulfate proteoglycans; ITGA5, integrin α5; MDM, monocyte-derived macrophages; RUNX1, Runt-related transcription factor 1; SPP1, secreted phosphoprotein 1; TNC, tenascin-C).

Exosomes secreted by glioma cells carry immunosuppressive molecules that further impair the activity of immune cells (221). For instance, mesenchymal subtype glioblastoma cells (MES-GBM) express the circular RNA circSDHAF2, which stabilizes integrin α5 (ITGA5) by promoting β4-galactosyltransferase-1 (B4GALT1)-mediated N-glycosylation. The stabilized ITGA5 protein is subsequently packaged into exosomes and delivered to monocyte derived macrophages (MDMs), where it activates the focal adhesion kinase/runt-related transcription factor 1 (FAK/RUNX1) axis to promote SPP1 transcription. This process induces the formation of an SPP1-high MDM subpopulation, thereby establishing a spatially specific immunosuppressive microenvironment (222) (**Figure 11**).

The brain extracellular matrix (ECM) contributes to brain tumorigenesis and metastasis. The stromal ECM of the brain parenchyma consists of collagens, laminins, and fibronectin, with its major constituents being large proteoglycans, glycoproteins, and glycosaminoglycans such as heparan sulfate proteoglycans (HSPGs) and hyaluronic acid (HA). These macromolecules accumulate in specialized niches including neural stem cell niches and vascular niches (223). HSPGs, which are abundantly produced within gliomas (224), serve as reservoirs for fibroblast growth factors (FGFs) and vascular endothelial growth factors (VEGFs). Additionally, the vessel-associated macromolecules tenascin-C (TNC) and periostin promote cancer cell survival (225–227). TNC further contributes to an immunosuppressive microenvironment by recruiting tumor-promoting immunosuppressive macrophages (228) and impeding T cell infiltration into the brain parenchyma (229) (**Figure 11**).

### 5.4 An integrated chemical–cellular suppressive architecture

The TME functions as a “fortress,” actively impeding, exhausting, or “subverting” immune cells through diverse mechanisms. Its core architecture can be summarized as three interrelated barriers—physical, chemical, and cellular (**Table 4**). The chemical and cellular barriers are considered here; the physical barrier, which is inseparable from the neurovascular interface, is treated in Section 5.2.

**Table 4:** Three-barrier immunosuppressive architecture of the GBM tumor microenvironment.

Barrier type	Mechanism	Key molecules	Effect on immune cells	References
Physical barrier	Aberrant vasculature; elevated interstitial fluid pressure; dense stromal ECM	Collagen, Hyaluronan, ICAM-1 ↓, VCAM-1 ↓	Immune cell trafficking ↓; infiltration into tumor depth ↓; homing impaired	(231, 250, 263)
Chemical barrier	Immune checkpoint upregulation	PD-L1, PD-1	T cell exhaustion and functional impairment	(230)
	Immunosuppressive cytokines	TGF-β, IL-10	Treg generation ↑; DC maturation ↓	(231, 232)
	Metabolic nutrient depletion	IDO (tryptophan), ARG1 (arginine)	T cell activation and proliferation ↓	(231)
	Hypoxia and acidosis (lactate accumulation)	Lactate, HIF-1α	Effector T cell and NK cell cytotoxicity ↓	(233, 234)
Cellular barrier	Immunosuppressive cell recruitment and reprogramming	GAMs/TAMs, Tregs, MDSCs, CAFs	Anti-tumor immunity ↓; angiogenesis ↑; tissue remodeling	(235–238)
	MHC-I downregulation and antigenic variation	MHC-I ↓	CD8^+^ CTL recognition and killing ↓	(239)

A chemical barrier is established through the expression or depletion of key signaling molecules. A primary mechanism is the upregulation of immune checkpoint molecules. Tumor cells and other components within the TME frequently overexpress ligands such as PD-L1. The engagement of PD-L1 with its receptor PD-1 on T cells drives T cell dysfunction, leading to a state of “exhaustion” or functional impairment (230). Further contributing to immunosuppression is the secretion of soluble inhibitory factors. TGF-β acts as a potent immunosuppressant and promotes the generation and activity of Tregs (231) (232). IL-10 inhibits the maturation and antigen-presenting function of dendritic cells, thereby fostering an immunosuppressive milieu (232). Metabolically, tumors deplete essential nutrients through enzymatic activity. High consumption of tryptophan (mediated by IDO) and arginine (mediated by arginase 1, ARG1) (231) starves infiltrating T cells of these amino acids, which are critical for their activation and proliferation. Finally, the dysregulated tumor metabolism creates a hostile physicochemical environment. Rapid tumor growth leads to hypoxia and the subsequent accumulation of lactate, resulting in extracellular acidosis. This hypoxic and acidic niche is selectively advantageous for tumor cell survival and the function of immunosuppressive cells, while simultaneously impairing the cytotoxic activity of effector T cells and NK cells (233, 234).

A cellular barrier is erected through the recruitment and reprogramming of immunosuppressive cell populations within the TME. Tregs are co-opted by tumors to actively suppress antitumor immunity. In the CNS, GAMs are frequently polarized towards immunosuppressive states, which lacks tumoricidal activity and instead secretes immunosuppressive cytokines, promotes angiogenesis, and facilitates tissue remodeling conducive to tumor progression (235, 236). Myeloid-derived suppressor cells (MDSCs) expand in the TME and directly inhibit the function of both T cells and natural killer (NK) cells (237). Cancer-associated fibroblasts (CAFs), beyond their role in constructing the physical stromal barrier, also contribute to the chemical barrier by secreting various immunosuppressive factors (238).Furthermore, tumor cell-intrinsic mechanisms reinforce this barrier. Tumor heterogeneity and ongoing evolution lead to antigenic variation and loss, enabling tumor cells to effectively “hide” from immune surveillance—a phenomenon akin to immunoediting. Additionally, many tumor cells downregulate or completely lose the expression of major histocompatibility complex class I (MHC-I) molecules, thereby diminishing their immunogenicity and impairing recognition and killing by CD8+ cytotoxic T lymphocytes (239). Beyond these intratumoural barriers, glioblastoma is uniquely constrained by the anatomical interfaces that regulate immune access to the CNS, which are considered in the following section.

## 6 The blood-brain barrier and neurovascular interface

### 6.1 Compartmentalization of CNS immune surveillance

In addition to microglia — a specialized population of brain-resident macrophages that execute key immune functions in the nervous system, such as rapid response to injury or infection (240) and secretion of cytokines and chemokines to modulate inflammatory responses (241)— the cerebral immune compartment also includes bone marrow‐derived macrophages (242). These are recruited from circulating monocytes into the brain microenvironment under conditions of disrupted homeostasis or pathology, where they subsequently differentiate (243). Dendritic cells further contribute to cerebral immunity through their potent antigen-presenting capacity, actively participating in a range of pathological contexts including brain infections (244), neurodegenerative diseases (245), and autoimmune disorders (246). During cerebral infection or inflammation, T lymphocytes, B lymphocytes, and monocytes can be recruited across the blood brain barrier via chemokine and cytokine signaling; once within the parenchyma, they secrete additional cytokines and chemokines to shape and amplify the local immune response (247–249).

It is generally accepted that in healthy individuals, immune cells within the CNS are predominantly localized to the meninges and the choroid plexus. The interface between the cerebral vasculature and the brain parenchyma is fortified by the blood–brain barrier (BBB), which imposes a stringent compartmentalization. Direct interactions between blood-borne components and CNS tissue are primarily restricted to the circumventricular organs (CVOs). Collectively, these four anatomical domains constitute the brain’s border regions, each of which is innervated (250). Certain cancer cells are capable of traversing the BBB—for instance, by disrupting endothelial junctional adhesion molecules such as JAM-B (251)—thereby evading the effects of chemotherapeutic agents that are otherwise impermeable to the BBB. Additional site-specific mediators that facilitate trans-BBB migration include prostaglandin-endoperoxide synthase 2 (PTGS2), heparin-binding EGF-like growth factor (HB-EGF), and ST6GALNAC5 (252).

In the context of intracranial neoplasms, peripheral immune cells are confronted with a dual barrier: prior to navigating the immunosuppressive tumor microenvironment, they must first surmount the BBB. Resident microglia and monocyte-derived macrophages within the CNS exhibit considerable phenotypic overlap (253–256). Under inflammatory conditions, the microglia-specific markers transmembrane protein 119 (TMEM119) and P2RY12 are downregulated (256–258), while infiltrating monocytes, upon entry into the brain parenchyma, may upregulate TMEM119 expression (256) (259–261), thereby complicating their distinction from resident microglia. Studies in murine models of colitis have demonstrated that expression of the fenestrae-associated protein plasmalemma vesicle-associated protein (PV1) in choroid plexus endothelial cells modulates vascular tone, permitting macrophage-mediated antigen capture during vasodilation while limiting inflammation upon vasoconstriction (262). Notably, PV1 deficiency results in anxiety-like behaviors in mice, underscoring a mechanistic link between peripheral inflammation and the onset of affective dysregulation (262).

### 6.2 The perivascular niche and vascular abnormality as a physical barrier

The physical barrier is primarily constituted by the aberrant tumor vasculature. To sustain rapid proliferation, tumors promote extensive neovascularization; however, these vessels are structurally disorganized, tortuous, and poorly perfused, resulting in inefficient blood flow and impeding the effective trafficking of immune cells to tumor sites (263). Elevated interstitial fluid pressure, caused by vascular leakage and accumulation of fluid within the tumor, further physically restricts immune cell infiltration into deeper tumor regions (231). Additionally, tumor cells and associated fibroblasts secrete excessive amounts of collagen, hyaluronan, and other extracellular matrix components, forming a dense stromal barrier that obstructs immune cell migration. The absence of key adhesion molecules—such as intercellular adhesion molecule 1 (ICAM-1) and vascular cell adhesion molecule 1 (VCAM-1)—on vascular endothelial cells further compromises the “homing” process, impairing immune cell adhesion and extravasation into the tumor parenchyma (250).

Moreover, intracranial tumors can give rise to neural stem cell (NSC)-like populations, which are capable of differentiating into endothelial-like cells (264). The perivascular niche, situated in close apposition to the BBB, serves as a reservoir for glioma stem cells (265), and this architectural arrangement is highly conducive to glioma dissemination.

### 6.3 Meningeal lymphatics and glymphatic drainage

Over the past decade, the cerebral lymphatic system—commonly referred to as the glymphatic system—has garnered increasing research attention. Anatomically, the glymphatic system is composed of the cerebrospinal fluid (CSF) compartments, cerebral arteries, astrocytic endfeet, and venous vasculature. In murine models, the dural sinuses and meningeal arteries have been shown to express lymphatic endothelial markers such as Lyve-1, VEGFR3, podoplanin, and Prox1, which are typically restricted to conventional lymphatic vessels. Notably, this system is contiguous with cervical lymph nodes, establishing a functional conduit between the CNS and peripheral lymphoid organs (266, 267). The glymphatic system also serves as a major niche for CNS-resident lymphocytes and harbors a dynamic repertoire of immune cells (38) (268–270), including adaptive subsets (CD4+ and CD8+T cells, B cells), innate lymphoid cells (e.g., NK cells), microglia, macrophages, neutrophils, monocytes, and dendritic cells. The phenotypic profiles of these populations are shaped by the local microenvironment and CSF-borne nutrients—such as cholesterol, thyroid hormones, and insulin-like growth factors—and often diverge from those of their peripheral counterparts (38) (270–273). Meningeal lymphatic vessels, situated within the dura mater (267) and defined by expression of VEGFR3, CCL21, and PROX1 (274), mediate the efflux of interstitial fluid, soluble proteins, antigens, and immune cells from the CNS to peripheral lymph nodes (275). Functional ablation of the meningeal lymphatic network in a murine model of multiple sclerosis attenuates T cell-mediated neuroinflammation, underscoring its role in propagating adaptive immune responses within the CNS (276).

## 7 Therapeutic opportunities

### 7.1 Overcoming the delivery barrier

Several complementary strategies have emerged to enhance drug delivery across the BBB in the context of CNS malignancies. Reversible and spatially confined BBB disruption can be achieved via intravascular administration of hyperosmotic agents such as mannitol or through advanced techniques including focused ultrasound in conjunction with microbubbles, thereby facilitating transient paracellular entry of therapeutic compounds (277, 278). Parallel efforts have focused on the rational design of BBB-penetrant agents, exemplified by “Trojan horse” constructs engineered to exploit endogenous transport systems—most notably glucose transporters—or through encapsulation of therapeutics within nanocarriers capable of receptor-mediated transcytosis (279, 280). An additional axis of investigation seeks to intercept the reciprocal interactions between neoplastic cells and BBB endothelial constituents, with the objective of abrogating tumor cell adhesion, migration, and trans endothelial migration, thereby preempting metastatic colonization of the brain parenchyma. In this paradigm, the therapeutic target shifts from the tumor itself to the molecular architecture and signaling integrity of the BBB.

Enhancement of glymphatic system function—proposed on the basis of rodent studies and, to date, not validated in patients with CNS tumors—achievable through optimization of sleep architecture, adoption of specific body positioning, modulation of systemic blood pressure, or pharmacologic intervention—may facilitate the clearance of metabolic waste and pro-inflammatory solutes from the tumor microenvironment, attenuate cerebral edema, and establish more permissive conditions for adjunctive therapies (281). Moreover, the rational design of therapeutic vaccines and immunotherapeutic regimens that exploit glymphatic-mediated antigen trafficking is at present a hypothesis rather than an established strategy (282–284); the most direct supporting evidence remains preclinical, in which AAV- or mRNA-driven VEGF-C expansion of meningeal lymphatics enhanced CD8+ T cell priming and synergized with checkpoint blockade in murine glioblastoma (285). A comprehensive understanding and strategic engagement of this fluid–immune interface may also potentiate the efficacy of immune checkpoint inhibitors and other immunomodulatory agents in the treatment of intracranial neoplasms (283). No clinical trial has yet tested glymphatic or meningeal lymphatic modulation as an adjunct to immunotherapy in patients with brain tumors, and the statements in this paragraph should accordingly be read as preclinical and hypothesis-generating.

### 7.2 Immunotherapy, engineered cell therapy and microenvironment-directed approaches

Combination strategies integrating established or emerging immune checkpoint inhibitors—such as those targeting PD-1, PD-L1, or CTLA-4—with ex vivo engineered cellular therapeutics, including chimeric antigen receptor T (CAR-T) cells, CAR-NK cells, or tumor vaccines, represent a promising avenue for enhancing CNS tumor targeting. The need for such combinations is underscored by the fact that checkpoint blockade alone has so far failed in this setting: in the randomised phase 3 CheckMate 143 trial, single-agent nivolumab did not improve overall survival over bevacizumab in 369 patients with first-recurrence glioblastoma (median 9.8 vs 10.0 months), although responses were more durable and exploratory analyses suggested benefit in MGMT-methylated, corticosteroid-free patients (286). In this context, engineered immune cells have been proposed as vehicles to deliver checkpoint-blocking antibodies or other immunomodulatory payloads across the blood–brain barrier (BBB); this concept remains preclinical. What has been demonstrated in patients is locoregional delivery that bypasses rather than crosses the BBB: in first-in-human phase 1 studies, intraventricular CARv3-TEAM-E T cells and intrathecal bivalent CART-EGFR-IL13Rα2 cells produced rapid radiographic responses in recurrent GBM, although these were transient in most participants (287, 288). Physical BBB opening has likewise reached early-phase testing, with a skull-implantable low-intensity pulsed ultrasound device transiently opening the BBB and raising peritumoral paclitaxel concentrations several-fold (289). Parallel approaches seek to remodel the tumor microenvironment through vascular normalization via anti-angiogenic agents, including VEGF inhibitors, which can potentiate immune effector infiltration. Additional strategies aim to neutralize or reprogram immunosuppressive stromal populations, including GAMs/TAMs, cancer-associated fibroblasts (CAFs), and MDSCs. Metabolic modulation via enzymatic inhibitors—such as IDO inhibitors—has also been pursued to alleviate microenvironmental immunosuppression. Furthermore, next-generation oncolytic viruses are being engineered not only to mediate direct cytolysis of malignant cells but also to reshape the tumor immune landscape, elicit antigen release, provoke local inflammation, and promote the recruitment and activation of endogenous and adoptively transferred immune effectors within the tumor bed. We've summarised various potential strategies and related evidences (**Table 5**).

**Table 5:** Evidence grading of the principal mechanisms discussed in this review.

Evidence category	Mechanism/claim	Strongest supporting evidence	References
Human evidence	GAMs form a major fraction of the GBM mass and their abundance tracks malignancy grade	Flow cytometry, scRNA-seq and CITE-seq of resected patient tumors	(127–129, 131, 167)
	GAMs occupy a continuum of activation states structured by ontogeny and spatial niche	scRNA-seq and CyTOF of newly diagnosed and recurrent human GBM, with murine validation	(129–131, 167)
	Tumor-infiltrating T cells in GBM upregulate PD-1 and CTLA-4 and acquire an exhausted phenotype	Immunophenotyping of patient tumor and blood samples	(179–181)
	B7-H3 (CD276) is expressed in most GBM specimens and largely absent from normal brain	IHC and western blot of surgical specimens and patient-derived neurospheres	(184–186)
	A CD73-high macrophage population is specific to GBM and persists after anti-PD-1	Mass cytometry and scRNA-seq across five human tumor types	(189, 190)
	The GBM microenvironment imposes physical, chemical and cellular barriers to immune effectors	Patient specimens: aberrant vasculature, ICAM-1/VCAM-1 loss, IDO–kynurenine axis, MHC-I downregulation	(214–217, 230, 231, 235, 239, 263)
*In vivo* evidence	Neuron–glioma synaptic signaling (NLGN-3, BDNF, IGF-1) and tumor microtubes promote proliferation and invasion	Xenograft and intravital imaging of synaptic input and GAP-43/Ttyh1-dependent microtube networks	(113, 116, 119)
	CSF-1R blockade reprograms GAMs and restrains glioma growth	Pharmacological CSF-1 pathway inhibition in murine glioma reduces pro-tumor markers and attenuates growth	(140, 157)
	TIM-3 or LAG-3 blockade augments anti-PD-1 efficacy	Murine GL261 glioma with or without radiotherapy; target expression confirmed in human GBM	(187, 188)
	VEGF-C expansion of meningeal lymphatics enables CD8+ priming and synergizes with checkpoint blockade	AAV- and mRNA-driven VEGF-C in murine glioblastoma	(285)
	Adrenergic signaling (ADRB2/ADRB3) promotes tumor progression and angiogenesis	Prostate, pancreatic and hepatic tumor models	(102–104, 106)
	Neuron-derived midkine establishes a neuron–immune–cancer axis acting on CD8+ T cells	Murine optic pathway glioma	(109)
	Reprogrammed Schwann cells build tracks that promote perineural invasion	Pancreatic and breast cancer models	(110, 112)
	Glymphatic clearance can be modulated by sleep, posture and pharmacological intervention	Rodent physiology	(281–283)
*In vitro* evidence	Glutamate acting on NMDAR/AMPAR activates CaMKII/MAPK–CREB and invasion programmes	Tumor cell lines, with xenograft confirmation for selected findings	(88–91)
	Autocrine or paracrine acetylcholine sustains tumor growth via CHRM1/3/4 and CHRNA5	Thyroid, gastric, prostate and cholangiocarcinoma cell lines	(92, 94–96)
	Tumor-derived GABA suppresses CD8+ T cells through GABA-B receptor and β-catenin	Colon cancer cells with supporting mouse model	(93)
	Neurotrophin signaling through p75NTR and TrkB/TrkC enhances glioma invasiveness	Glioma cell lines and invasion assays	(105)
Therapeutic validation	Locoregional CAR-T delivery produces rapid radiographic responses in recurrent GBM	Phase 1: intraventricular CARv3-TEAM-E (n = 3); intrathecal CART-EGFR-IL13Rα2 (n = 6)	(287, 288)
	Ultrasound-mediated BBB opening increases intratumoural drug exposure	Phase 1: skull-implantable LIPU-MB device, 4–6-fold higher peritumoral paclitaxel	(289)
	Single-agent PD-1 blockade in unselected recurrent GBM	Randomized phase 3 (CheckMate 143, n = 369): no overall survival benefit vs bevacizumab	(286)

## 8 Conclusion and future perspectives

The nervous and immune systems, once considered functionally autonomous, are now recognized as deeply intertwined networks that co-regulate homeostasis, disease progression, and therapeutic response within the CNS. In this narrative review, we have delineated the cellular and molecular architecture underlying this bidirectional communication — from the receptor repertoires of neurons, astrocytes, microglia and oligodendrocytes (Section 1), through the neural inputs that drive tumor growth and the reciprocal tumor-to-neuron signals they provoke (Section 2), to the remodeling of glial and myeloid populations (Section 3), the innate and adaptive immune suppression that results (Section 4), the neurovascular interfaces that restrict immune access (Section 5), and finally the therapeutic opportunities arising at each of these levels (Section 6). Together, these lines of evidence reveal that neuroimmune communication is not a simple linear pathway but a network of feedforward and feedback loops that dynamically reshapes the CNS landscape in both neurological and neoplastic diseases.

Despite substantial progress, several critical gaps remain. (1) Most mechanistic insights into neuron–immune–tumor interactions derive from *in vitro* co-culture systems or murine models; whether these findings faithfully translate to the human CNS, with its distinct anatomical compartmentalization, tightly regulated rather than absent immune surveillance—operating through meningeal lymphatic drainage to the deep cervical lymph nodes, the perivascular and choroid plexus interfaces, and continuous trafficking of peripheral immune cells (14, 285)—and cellular heterogeneity, remains to be rigorously validated. (2) The temporal dynamics of neuroimmune crosstalk across disease stages are poorly understood. For instance, it is unclear how the balance between neuroprotective and neurotoxic glial phenotypes shifts during the transition from low-grade to high-grade gliomas, or from acute neuroinflammation to chronic neurodegeneration. Longitudinal single-cell and spatial transcriptomic studies will be essential to resolve these questions. (3) While individual signaling axes (e.g., NMDAR–CaMKII–CREB, ADRB2–MAPK–ERK, CSF-1–CSF-1R) have been characterized in isolation, the systems-level integration of these pathways, including their synergistic, antagonistic, and compensatory interactions, remains largely unexplored. Computational modeling and multi-omics network analyses could provide the integrative framework needed to predict emergent behaviors of this complex system. (4) The role of peripheral immune cells that infiltrate the CNS under pathological conditions, and how their interactions with resident neural and glial populations differ from those of CNS-native immune cells, warrants further investigation.

In conclusion, neuroimmune communication in the CNS is a therapeutically consequential interface whose mechanisms are unevenly established: neuron–glioma glutamatergic signaling, GAM heterogeneity and checkpoint-mediated T cell dysfunction are supported by human data, whereas glymphatic modulation and neuro-immune engineering remain preclinical. Progress will require mechanistic dissection at the molecular and cellular levels together with integrative approaches bridging neuroscience, immunology and oncology, with the aim of yielding diagnostic biomarkers and therapies that are validated in patients rather than in models alone.

## Glossary

AMPAR: α-amino-3-hydroxy-5-methyl-4-isoxazolepropionic acid receptor
ADGRG1: adhesion G protein-coupled receptor
ADNF: activity-dependent neuroprotective factor
ADRB2: beta-2 adrenergic receptors
AHR: aryl hydrocarbon receptor
AKT: protein kinase B
Arg: arginase
Atg4: autophagy related 14
B4GALT1: β4-galactosyltransferase-1
BBB: blood-brain barrier
BDNF: brain-derived neurotrophic factor
CAFs: Cancer-associated fibroblasts
CaMKII: calmodulin-dependent protein kinase II
CCL: C-C motif chemokine ligand
CCR: C-C chemokine receptor
cGAMP: cyclic GMP-AMP
cGAS-STING: cyclic GMP-AMP synthase-stimulator of interferon genes
CGRP: calcitonin gene-related peptide
CHRM1: cholinergic receptor muscarinic 1
CHRNA5: α5 nicotinic acetylcholine receptor subunit
CICR: calcium-induced calcium release
CircSDHAF2: circular RNA succinate dehydrogenase complex assembly factor 2
CNS: central nervous system
CREB: cAMP response element-binding protein
CSF: cerebrospinal fluid
CSF-1: colony-stimulating factor 1
CSPGs: chondroitin sulfate proteoglycans
CTLA-4: cytotoxic T-lymphocyte–associated protein 4
CVOs: circumventricular organs
CX3CL1: C-X3-C motif chemokine ligand 1
CXCL: C-X-C motif chemokine ligand
CXCR: C-X-C chemokine receptor
DCs: dendritic cells
DDR1: discoidin domain receptor 1
EAE: experimental autoimmune encephalomyelitis
ECM: extracellular matrix
EGF: epidermal growth factor
EGFR: epidermal growth factor receptor
EMT: epithelial-mesenchymal transition
Epac: exchange protein activated by cAMP
EPOR: erythropoietin receptor
ERK: extracellular signal-regulated kinase
ETV: ETS translocation variant 1
FAK: focal adhesion kinase
FAK/RUNX1: focal adhesion kinase/runt-related transcription factor 1
FGF: fibroblast growth factors
FMRP: Fragile X Messenger Ribonucleoprotein
FN: fibronectin
GABA: γ-aminobutyric acid
GAMs: glioma-associated macrophages/microglias
GAP-43: growth-associated protein 43
GBM: glioblastoma multiforme
G-CSF: granulocyte-colony stimulating factor
GIST: gastrointestinal stromal tumors
GKAP: guanylate kinase-associated protein
GM-CSF: granulocyte-macrophage colony-stimulating factor
GPCR: G protein-coupled receptor
GSK-3β: glycogen synthase kinase 3β
HA: hyaluronic acid
HB-EGF: heparin-binding EGF-like growth factor
HCN2: hyperpolarization-activated cyclic nucleotide–gated
HGF: hepatocyte growth factor
HIF: hypoxia-inducible factor
HK2: hexokinase 2
HMGB1: high-mobility group box
HSF1: heat shock factor 1
HSPG: Runt-related transcription factor 1
hTERT: human telomerase reverse transcriptase
IBP-2: insulin-like growth factor-binding protein 2
ICAM: intercellular adhesion molecule
IDO1: indoleamine 2,3-dioxygenase 1
IFN: interferon
IGF: insulin-like growth factor
iGluRs: ionotropic glutamate receptors
IL: interleukin
iNOS: inducible nitric oxide synthase
IRF3: interferon regulatory factor 3
ITGA5: integrin α5
JKA: Janus kinase
JNK: Jun N-terminal kinase
kyn: L-kynurenine
LAG-3: lymphocyte activation gene-3
LOX: lysyl oxidase
MAPK: mitogen-activated protein kinase
MCP: monocyte chemoattractant protein
MDK: Midkine
MDMs: monocyte-derived macrophages
MDSC: myeloid-derived suppressor cells
MEK: mitogen-activated extracellular signal-regulated kinase
MES-GBM: mesenchymal subtype glioblastoma cells
METTL3: methyltransferase-like 3
mGluRs: metabotropic glutamate receptors
MHC-I: histocompatibility complex class I
MIC-1: macrophage inhibitory cytokine-1
MMP-2: matrix metallopeptidase-2
mTOR: mammalian target of rapamycin
MYDGF: myeloid-derived growth factor
nAChRs: nicotinic acetylcholine receptors
NET: neutrophil extracellular trap
NF-κB: nuclear factor kappa-B
NG2: nerve/glial antigen 2
NGF: nerve growth factor
NK: natural killer
NLGN-3: neuroligin-3
NMDAR: N-methyl-D-aspartate receptor
NO: nitric oxide
NSC: neural stem cell
NT-4: neurotrophin-4
OPCs: oligodendrocyte progenitor cells
p75NTR: p75 neurotrophin receptor
PAK: p21-activated kinase
PD-1: programmed cell death 1
PD-L1: programmed cell death 1 ligand 1
PGE-2: prostaglandin E2
PI3K: phosphatidylinositol 3-kinase
PKA: protein kinase A
PNI: perineural invasion
PP2A: protein phosphatase 2A
PROX1: prospero homeobox 1
PTEN: phosphatase and tensin homolog
PTGS2: prostaglandin-endoperoxide synthase 2
PYK2: proline-rich tyrosine kinase 2
RAS: rat sarcoma virus
ROCK: Rho-associated coiled-coil containing protein kinase
SDF-1: stromal cell-derived factor 1
SP-1: specificity protein 1
SPARC: secreted protein acidic and rich in cysteine
SPARCL-1: SPARC-like protein 1
SPP-1: secreted phosphoprotein 1
Src: Src proto-oncogene
ST6GALANC5: ST6 N-acetylgalactosaminide alpha-2,6-sialyltransferase 5
STAT3: signal transducer and activator of transcription 3
TAK-1: TGFβ-activated kinase 1
TAMs: tumor-associated macrophages
TASTs: tumor-activated Schwann cell trajectories
TBI: traumatic brain injury
TCR: T cell receptor
TGF: tumor growth factor
Thbs1/2: thrombospondins
TIM-3: T cell immunoglobulin and mucin domain-3
TIMP-2: tissue inhibitor of metalloproteinase 2
TME: tumor microenvironment
TMEM119: transmembrane protein 119
TNC: tenascin-C
TNF: tumor necrosis factor
Treg: regulatory T cell
Trk: tropomyosin receptor kinase
Ttyh1: tweety-homolog 1
VCAM: vascular cell adhesion molecule
VEGF: vascular endothelial growth factor
VGIC: voltage-gated ion channels
VPS34: vacuolar protein sorting 34
YAP1: Yes-associated protein 1.
